# Optimal individualized decision rules from a multi-arm trial: A comparison of methods and an application to tailoring inter-donation intervals among blood donors in the UK

**DOI:** 10.1177/0962280220920669

**Published:** 2020-05-08

**Authors:** Yuejia Xu, Angela M Wood, Michael J Sweeting, David J Roberts, Brian DM Tom

**Affiliations:** 1 MRC Biostatistics Unit, University of Cambridge, Cambridge, UK; 2 Cardiovascular Epidemiology Unit, University of Cambridge, Cambridge, UK; 3 NIHR Blood and Transplant Research Unit in Donor Health and Genomics, Cambridge, UK; 4 Department of Health Sciences, University of Leicester, Leicester, UK; 5 BRC Haematology Theme and Radcliffe Department of Medicine, University of Oxford, Oxford, UK; 6 National Health Service Blood and Transplant, Oxford, UK

**Keywords:** Precision medicine, individualized treatment rule, multi-arm trial, utility function, blood donation

## Abstract

There is a growing interest in precision medicine where individual heterogeneity is incorporated into decision-making and treatments are tailored to individuals to provide better healthcare. One important aspect of precision medicine is the estimation of the optimal individualized treatment rule (ITR) that optimizes the expected outcome. Most methods developed for this purpose are restricted to the setting with two treatments, while clinical studies with more than two treatments are common in practice. In this work, we summarize methods to estimate the optimal ITR in the multi-arm setting and compare their performance in large-scale clinical trials via simulation studies. We then illustrate their utilities with a case study using the data from the INTERVAL trial, which randomly assigned over 20,000 male blood donors from England to one of the three inter-donation intervals (12-week, 10-week, and eight-week) over two years. We estimate the optimal individualized donation strategies under three different objectives. Our findings are fairly consistent across five different approaches that are applied: when we target the maximization of the total units of blood collected, almost all donors are assigned to the eight-week inter-donation interval, whereas if we aim at minimizing the low hemoglobin deferral rates, almost all donors are assigned to donate every 12 weeks. However, when the goal is to maximize the utility score that “discounts” the total units of blood collected by the incidences of low hemoglobin deferrals, we observe some heterogeneity in the optimal inter-donation interval across donors and the optimal donor assignment strategy is highly dependent on the trade-off parameter in the utility function.

## 1 Introduction

Precision medicine is a rapidly expanding field in the new era of healthcare with major advancements having been made in technologies for collecting patient-level data and better characterizing each individual patient. The goal of precision medicine is to improve patient outcomes by tailoring treatment selection based on observed patient characteristics, for example, demographic information, clinical and laboratory measurements, medical history, and genetic data. In clinical practice, it is well recognized that responses to treatments can vary substantially due to patient heterogeneity. Therefore, the treatment that is regarded as the best for a patient with one set of characteristics might not be the best for another, and the traditional “one-size-fits-all” approach does not lead to optimal clinical outcomes in many cases. In light of this, individualized, evidence-based clinical decision-making strategies that account for such heterogeneity are considered more desirable and are gaining much popularity in medical research.

One important aspect of precision medicine is the estimation of the optimal individualized treatment rule (ITR) by mapping patient information onto the set of treatment options. There has been a considerable amount of literature focusing on this matter. We refer readers to Lipkovich et al.^1^ and Kosorok and Laber^2^ for a comprehensive review of existing methods.

Clinical trials with multiple treatment arms are common in practice.^3^ However, most existing statistical methods for estimating the optimal ITR were developed for binary treatment arm settings, and methods that directly aggregate multiple pairwise comparison results such as one-versus-rest or one-versus-one can be problematic: there are situations where the two-way preferences may be non-transitive and thus a consensus on the final decision of the original multi-category comparison problem cannot be reached. In addition, this may result in suboptimal decisions (simulated examples demonstrating the problem can be found in Lou et al.^4^ and Zhou et al.^5^). For example, in the context of multi-class classification, it has been shown that the optimal solution obtained from the one-versus-rest approach can be different from the Bayes decision rule when there is no dominating class.^6^ In this paper, we describe several methodological options that can be used to identify the optimal ITR in clinical trials with more than two treatment arms and are computationally feasible for large-scale trials, including *l*_1_-penalized least squares,^7^ adaptive contrast weighted learning,^8^ direct learning,^9^ and a Bayesian approach that is based on Bayesian additive regression trees.^10^ Our motivating example, the INTERVAL trial,^11,12^ had more than 20,000 observations on each gender, and we are in particular interested in the performance of these methods on large datasets similar in size to the INTERVAL trial. However, to our knowledge, there is a lack of studies evaluating their performance in datasets with sample sizes larger than 2000. To fill this gap, we compare the aforementioned multi-arm ITR estimation methods in large-scale trials via simulation studies. We then conduct a case study, applying these methods to the data from the INTERVAL trial to estimate the optimal inter-donation interval among three possible options (12-week, 10-week, and eight-week) for male donors. We estimate the proportion of blood donors allocated to each inter-donation interval and quantify the gain (or loss) in outcomes when assigning donors according to the optimal ITRs inferred using these methods under three different targets: (1) maximizing the total units of whole blood collected by the blood service, (2) minimizing the risk of deferral for low hemoglobin (Hb), and (3) maximizing a utility score that seeks to balance the amount of blood collected against the number of deferrals for low Hb. We note from the INTERVAL trial that only considering maximizing the blood collection (target 1) or minimizing deferrals for low Hb (target 2) results in donor assignment strategies that are not particularly surprising and quite “polar” in nature.^12^ The purpose of investigating these two targets by applying precision medicine-based approaches is to examine in extreme cases where the universal rule (i.e. non-personalized strategy) should lead to nearly optimal outcomes, whether ITRs estimated using precision medicine-based methods are “almost non-personalized” and assign almost all donors to the marginally best inter-donation interval option, or instead “falsely” discover a fair amount of heterogeneity in the optimal inter-donation interval across different donors.^13^ To our knowledge, this aspect has been rarely explored in the area of precision medicine as most researchers begin by assuming that patient subgroups exist and different patients would benefit from different treatment strategies. For male blood donors in the INTERVAL trial, results suggest that different statistical methods to estimate the optimal ITR lead to fairly similar recommendations on the optimal donation strategies: optimal ITRs estimated under the first two targets are very close to “one-size-fits-all” strategies, while optimal ITRs estimated under the third target suggest some heterogeneity in different blood donors’ optimal inter-donation intervals. In addition, the optimal ITR is highly dependent on how the utility score is constructed, i.e. the trade-off between the blood collection and the deferrals for low Hb.

The rest of the paper is organized as follows. In Section 2, we introduce the INTERVAL trial. In Section 3, we discuss the statistical framework and methods for estimating the optimal ITR when the treatment is binary. In Section 4, we review a selection of methods for estimating the optimal ITR when more than two treatment options are available. In Section 5, we conduct simulation studies to evaluate the performance of the multi-arm ITR estimation methods described in Section 4 under different scenarios. Section 6 presents results for an application of these methods to the INTERVAL trial dataset. We conclude with the discussion in Section 7.

## 2 Motivating example: the INTERVAL trial

Limits on the frequency of whole blood donation exist primarily to safeguard donor health. However, there is substantial variation across blood services in the maximum frequency of donations allowed. The National Health Service Blood and Transplant (NHSBT) in England currently allows a minimum inter-donation interval of 12 weeks for males and 16 weeks for females, with shorter inter-donation intervals used in other countries (e.g. the USA and France).^14–16^ INTERVAL was the first randomized trial to evaluate the efficiency and safety of varying the frequency of whole blood donation. It randomly assigned over 45,000 blood donors recruited across England to different inter-donation intervals (8, 10, and 12 weeks for men, and 12, 14, and 16 weeks for women) over a period of two  years. During that time, there was a substantial increase in the amount of blood collected by reducing the inter-donation intervals without any detectable effects on overall quality of life, physical activity, or cognitive function.^11,12,17^ However, increased donation frequency resulted in a greater number of deferrals (temporary suspension of donors from giving blood) due to low Hb, lower average Hb and ferritin concentrations, and more self-reported symptoms.

INTERVAL trial participants were well characterized at baseline,^17^ providing an opportunity to further explore personalized donation strategies whereby the amount of blood collected is optimized while controlling for the number of deferrals for low Hb. We expect differential relationships between the amount of blood collected and the number of deferrals for low Hb by donors’ individual characteristics. For example, young, female donors with low body mass index may be less able to donate more frequently without a rise in the number of deferrals for low Hb, whereas donors with a long, successful donation history may be able to donate more frequently with no increase in the number of deferrals for low Hb.^11^ We compare the personalized donation strategies against assignments of the same inter-donation interval for all donors (current clinical practice).

## 3 Preliminaries

### 3.1 Notations and statistical frameworks

We consider a clinical trial where *n* subjects are sampled from a population of interest. Let *Y* be the outcome of interest, A∈A={1,…,K} denote the treatment assignment, and X∈X be a *p*-dimensional covariate (feature) vector. Without loss of generality, we assume a larger value of *Y* is preferred. We observe the triplet $\left(Y_{i},X_{i},A_{i}\right)$ , for i=1,…,n, which are independent and identically distributed across *i*. The individualized treatment rule (ITR), denoted by D , is a map from the space of feature variables, X , to the domain of treatment assignments, A . We assume the propensity score P(A=a|X) is bounded strictly away from 0, i.e. P(A=a|X=x)>0 for all pairs (x,a)∈X×A . In clinical trials, the no unmeasured confounders assumption is always satisfied (for the intention-to-treat analysis).^18^ Assuming that the consistency assumption holds,^19^ the “value function” associated with the treatment rule D can be derived as

(1) $$V\left(D\right)=E\left\{\frac{I\left(A=D\left(X\right)\right)}{P\left(A|X\right)}Y\right\}$$

where E(.) is the expectation and I(.) is the indicator function.^7^ The value function V(D) is the expected value of the outcome *Y* had the regime D been applied to the given population. Our goal is to find the optimal ITR, $D^{*}$ , that maximizes the value function under D , i.e.

(2) $$D^{*}=\underset{D}{\text{arg}\;\text{max}}V\left(D\right)\text{=}\underset{D}{\text{arg}\;\text{max}}E\left\{\frac{I(A=D(X))}{P(A|X)}Y\right\}$$

### 3.2 Estimation of the optimal ITR when K = 2

Many statistical methods have been proposed to estimate the optimal ITR in the case with two treatments (*K *=* *2), including indirect and direct methods.

Q-learning and A-learning are two main examples of indirect estimation methods, where the estimation of the optimal regime relies on modelling conditional mean outcomes (Q-learning) or modelling treatment contrasts (A-learning). The optimal ITR is then inferred (indirectly) based on the predicted means from the posited model. In the Q-learning paradigm, a model including treatment-covariate interactions is postulated for E(Y|X,A=a) . It can be shown from equation (2) that $D^{*}(x)=\text{arg}\;{\text{max}}_{\text{a}}E(\mathrm{Y|}X\text{=}x,A\text{=a})$ , and thus the decision follows naturally by maximizing the outcome from the fitted model, i.e. $\hat{D^{*}}\left(x\right)=\text{arg}\;{\text{max}}_{\text{a}}\hat{E}\left(Y|X\text{=}x,A=a\right)$ .^20^ Alternatively, A-learning methods posit a model on the contrast function C(x) between two treatment options, and the final decision is based on the sign of the estimated contrast $\hat{C}(x)$ .^21^ Compared to Q-learning, A-learning avoids the modelling of marginal covariate effects. Other indirect methods include G-estimation,^22^ regret regression,^23^ and weighted ordinary least squares.^24^ Those indirect approaches focus on building a prediction model for either the conditional mean outcome or the treatment contrast and they target prediction accuracy instead of the direct maximization of the value function. We also note that methods such as G-estimation and weighted ordinary least squares are doubly-robust. In randomized trials, the treatment assignment model is known by design, and thus these methods can be preferable to Q-learning as they offer protection against the misspecification of the outcome model.^24,25^

Alternatively, Zhao et al.^26^ proposed a direct approach, the outcome weighted learning (OWL), to estimate the optimal ITR (directly) by maximizing the value function V(D) rather than (indirectly) by inverting estimates from prediction models. Since equation (2) indicates that $D^{*}=\text{arg}\;{\text{min}}_{D}E\left\{I\left(A\neq D\left(X\right)\right)Y/P\left(A|X\right)\right\}$ , Zhao et al.^26^ reformulated the problem of maximizing the value function into a weighted classification problem (with the aim of minimizing the weighted misclassification error), where they classified *A* based on **X** and weighted the misclassification by Y/P(A|X) . This could then be solved using support vector machines (SVM). To further improve the performance of OWL, Zhou et al.^27^ and Liu et al.^28^ introduced residual weighted learning (RWL) and augmented outcome weighted learning (AOL), respectively, by using different weights in the objective function.

Most of the aforementioned methods, except for Q-learning, were developed for the case of two treatments. A direct extension to the multi-arm setting by combining pairwise decision rules using methods such as one-versus-rest or one-versus-one may lead to suboptimal results.^5,6^

## 4 Review of a selection of methods for *K *>* *2

Recently, some new methods have been proposed to deal with the case of more than two treatments. In this section, we review (in publication order) a selection of approaches that can be used to estimate the optimal ITR in multi-arm trials and scale well for large datasets similar in size to the INTERVAL trial, including *l*_1_-penalized least squares,^7^ adaptive contrast weighted learning,^8^ direct learning,^9^ and a Bayesian approach that is based on Bayesian additive regression trees.^10^ We note that there are other recently proposed methods for learning the optimal ITR with multiple treatment options that we do not discuss in detail in this paper, for example, the multi-treatment outcome weighted learning,^4^ and the sequential outcome weighted multicategory learning,^5^ since they involve solving weighted support vector machine problems, which can impose high computational costs when applied to large datasets.^29^

### 4.1 l_1_-Penalized least squares (indirect method)

Qian and Murphy^7^ proposed the two-stage model-based *l*_1_-penalized least squares (*l*_1_-PLS) method. The conditional mean response was first estimated using *l*_1_-PLS with a rich linear model to guard against over-fitting and then the estimated means under different treatments were used to derive the optimal ITR. Specifically, we fit the model for E(Y|X,A) using the basis function (1,X,A,XA) , in which we use *K* – 1 dummy variables to replace *A*. The decision can be derived as $\hat{D^{*}}\left(x\right)=\text{arg}\;{\text{max}}_{a\in \{\text{1},\ldots ,\text{K}\}}\hat{E}\left(Y|X\text{ = }x,A=a\right)$ .

**X** in the INTERVAL trial dataset contains several nominal categorical covariates. In order to avoid the over-selection of variables with many categories and ensure that all dummy variables encoding the same categorical covariate are either included or excluded from the model simultaneously, we use group LASSO (GL) which encourages sparsity at the factor level for variable selection.^30,31^

Another variable selection approach that is of interest in this context is hierarchical group LASSO (HGL). HGL was designed for learning pairwise interactions in regression models that satisfy strong hierarchy, i.e. if the coefficient associated with the interaction term is estimated to be non-zero, then its two associated main effects also have non-zero estimated coefficients. The assumption underlying HGL is that covariates that predict treatment effect heterogeneity (included as interactions) are also prognostic (included in main effects).

As has been pointed out by Gunter et al.,^31^ maintaining hierarchy in variable selection avoids finding spurious interactions that may appear due to the exclusion of important main effect terms. The HGL method proposed by Lim and Hastie^32^ picks out only main effects if the truth has no interactions, but when interactions truly exist, it allows the discovery of important interaction terms despite their weak effects by regularizing coefficients of main effects using the “glinternet” penalty. We can embed HGL in the first step of *l*_1_-PLS to identify important treatment-covariate interactions. For ease of interpretation, we restrict the search space to be all possible two-way interactions between treatment *A* and covariates **X**.

As an aside, there are two types of interactions: quantitative interactions and qualitative interactions.^1^ A quantitative interaction between *A* and **X** refers to the situation where the magnitude of the effect of *A* on the outcome *Y* depends on **X** but the direction of this effect is consistent for all possible values of **X**, whereas a qualitative interaction between *A* and **X** indicates that both the direction and the magnitude of *A*’s effect on the outcome *Y* can depend on **X** (Supplementary Figure 1 in the online supplemental materials Appendix A). GL and HGL interaction selection procedures are not able to automatically distinguish between qualitative and quantitative interactions. Therefore, further assessment of interactions (e.g. using the Gail-Simon test^33^) is required to determine whether the selected interactions are useful for achieving specific aims (e.g. the aim can either be to detect a qualitative difference in treatment effects, i.e. crossover treatment effect heterogeneity, or to detect a quantitative difference in treatment effects that informs the identification of subgroups with elevated response to a given treatment, i.e. non-crossover treatment effect heterogeneity) in the context of precision medicine.

One limitation of *l*_1_-PLS is that the correct inference on the optimal ITR depends on a correctly specified outcome model, while the postulated outcome model is prone to misspecification (especially when **X** is high-dimensional) and thus may result in suboptimal ITR. Unlike tree-based methods, which can capture more general forms of interactions, *l*_1_-PLS is restricted to searching for additive-type interactions. However, the true relationship between covariates, treatment assignment, and the outcome is usually complicated in practice, and the underlying interaction structures can be much more flexible than additive. Another issue with this approach is the interference between main effects and treatment-covariate interactions, which may impair the search for the optimal ITR. In most cases, the variability in the outcome is predominantly explained by main effects rather than interactions. Therefore, interaction effects can be overlooked due to their small predictive ability when the method focuses on prediction.^34–36^

### 4.2 Adaptive contrast weighted learning (direct method)

Adaptive contrast weighted learning (ACWL) was proposed by Tao and Wang^8^ to estimate the optimal ITR in the multi-arm setting. They constructed doubly-robust semi-parametric regression-based contrasts^37^ with the adaptation of treatment effect orderings, and the adaptive contrasts simplified the optimization problem with multiple treatment comparisons into a weighted classification problem.

Let $\mu_{a}(X)$ denote the conditional mean E(Y|X,A=a) . Tao and Wang,^8^ following Zhang et al.,^37^ employed the doubly-robust augmented inverse probability weighted estimator (AIPWE) for $\mu_{a}(X)$ . Let $\mu_{\left(1\right)}(X)\leq \ldots \leq \mu_{\left(K\right)}(X)$ be the order statistics of $\mu_{1}(X),\ldots ,\mu_{K}(X)$ , and *l_a_* be the treatment effect order with $\mu_{\left(a\right)}(X)=\mu_{l_{a}}(X)$ . It follows that

(3) $$\begin{array}{l} D^{*}(X)=\underset{D}{\text{arg}\;\text{max}}\sum_{a=1}^{K}E[\mu_{\left(a\right)}(X)I\{D(X)=l_{a}(X)\}]\\ =\underset{D}{\text{arg}\;\text{max}}\sum_{a=1}^{K\mathrm{-1}}E[\{\mu_{\left(K\right)}(X)\text{-}\mu_{\left(a\right)}(X)\}I\{D(X)=l_{a}(X)\}] \end{array}$$

The optimal ITR derived from equation (3) can be interpreted as the rule that minimizes the expected loss in the outcome due to suboptimal treatments in the entire population of interest, i.e. the ITR classifies as many subjects as possible to their corresponding optimal treatment *l_K_* and puts more “penalties” on subjects with larger contrasts. In practice, it can be challenging to utilize all *K* − 1 contrasts as weights to classify treatment. Tao and Wang^8^ addressed this by constructing the lower and upper bounds of the expected loss in the outcome due to suboptimal treatments as follows

(4) $$\begin{array}{l} \sum_{a=1}^{K-1}E[\{\mu_{\left(K\right)}(X)-\mu_{\left(a\right)}(X)\}I\{D(X)=l_{a}(X)\}]\\ \;\geq \sum_{a=1}^{K-1}E[\{\mu_{\left(K\right)}(X)-\mu_{\left(K-1\right)}(X)\}I\{D(X)=l_{a}(X)\}]\\ =E[C_{1}(X)I\{D(X)\neq \underset{\text{optimal}}{\underset{︸}{l_{K}(X)}}\}] \end{array}$$

and

(5) $$\begin{array}{l} \sum_{a=1}^{K-1}E[\{\mu_{\left(K\right)}(X)-\mu_{\left(a\right)}(X)\}I\{D(X)=l_{a}(X)\}]\\ \;\leq \sum_{a=1}^{K-1}E[\{\mu_{\left(K\right)}(X)-\mu_{\left(1\right)}(X)\}I\{D(X)=l_{a}(X)\}]\\ =E[C_{2}(X)I\{D(X)\neq \underset{\text{optimal}}{\underset{︸}{l_{K}(X)}}\}] \end{array}$$

where $C_{1}(X)=\mu_{\left(K\right)}(X)-\mu_{\left(K-1\right)}(X)$ and $C_{2}(X)=\mu_{\left(K\right)}(X)-\mu_{\left(1\right)}(X)$ . In the least conservative case where suboptimal treatments only lead to the minimal expected loss in the outcome

(6) $$D^{*}(X)=\underset{D}{\text{arg}\;\text{min}}E[C_{\text{1}}(X)I\{D(X)\neq l_{K}(X)\}]$$

while in the most conservative case where suboptimal treatments lead to the maximal expected loss in the outcome

(7) $$D^{*}(X)=\underset{D}{\text{arg}\;\text{min}}E[C_{\text{2}}(X)I\{D(X)\neq l_{K}(X)\}]$$

The optimal ITR estimated using equation (6) or (7) minimizes the lower or upper bounds of the expected loss in the outcome due to suboptimal treatments over the entire population, respectively. By optimizing these bounds, the optimization problem with multiple treatment comparisons is simplified to a weighted classification problem, which can be solved by classification techniques, such as classification and regression trees (CART).

ACWL is flexible and robust. However, additional uncertainties may be induced through the introduction of the “ordering labels”. Therefore, using adaptive contrasts may not be the most efficient way to avoid the multiple treatment comparison issue.

### 4.3 Direct learning (direct method)

Qi and Liu^9^ developed the direct learning (D-learning) approach that uses regression methods to directly estimate the optimal ITR. The advantage of D-learning over Q-learning is that D-learning directly targets the maximization of the value function, thus avoiding the need to model main effects.

In the binary arm case (treatment *A* is encoded as −1 or 1)

(8) $$\begin{array}{c} D^{*}(X)=\underset{D}{\text{arg}\;\text{max}}V(D)=\text{sign}[E(Y|X,A=1)-E(Y|X,A=-1)]\\ =\text{sign}[E\{\frac{YA}{P(A|X)}|X\}]:=\text{sign}\left\{f^{*}(X)\right\} \end{array}$$

where $f^{*}(X)$ is the optimal decision function.^9^ According to equation (8), $f^{*}(X)$ can be estimated using the fact that $f^{*}\left(X\right)=E\left\{\frac{YA}{P\left(A|X\right)}|X\right\}$ and Qi and Liu^9^ showed that $f^{*}\left(X\right)\in \text{arg}\;{\text{min}}_{f}E\left[{\left\{2YA-f(X)\right\}}^{\text{2}}/P(A\text{|}X)\right]$ . We can estimate $f^{*}(X)$ using regression methods for either linear or nonlinear decision rules. For example, suppose the decision function is linear and $f(X)=X^{T}\beta$ . Then β can be estimated using the ordinary weighted least squares (or LASSO in the high-dimensional case). The estimated optimal linear decision function is ${\;}^{\hat{f^{*}}}(X)=X^{T}\hat{\beta }$ , and the estimated optimal ITR $\hat{D^{*}}(X)$ is the sign of ${\;}^{\hat{f^{*}}}(X)$ .

D-learning was extended to the case with more than two treatments. Qi and Liu^9^ showed that the optimal ITR in the multi-arm setting can be written as

(9) $$\begin{array}{l} D^{*}\left(X\right)=\underset{a\in \{1,\ldots ,K\}}{\text{arg}\;\text{max}}\sum_{i\neq a}^{K}E\left\{\frac{{YA}_{\text{ai}}}{P\left(A_{ai}\text{|}X\right)}|X,A=a\text{ or }i\right\}\\ :=\underset{a\in \{\text{1},\ldots ,K\}}{\text{arg}\;\text{max}}\sum_{i\neq a}^{K}\;{f\;}_{ai}^{\text{*}}\left(X\right) \end{array}$$

where $A_{ai}\in \{-1,1\}$ denotes a binary treatment indicator for whether a patient is on treatment *i* ( $A_{ai}=-1$ ) or on treatment *a* (*A_ai_* = 1) for i≠a , and $f_{ai}^{*}(X)$ denotes the optimal decision function between treatment *a* and *i*. Each pairwise decision function $f_{ai}^{*}(X)$ , for a,i=1,…,K,i≠a , can be estimated using regression methods as in the binary setting.

We note that even though multi-category D-learning builds on multiple pairwise comparisons, it is different from the one-versus-one approach^38^ in that one-versus-one aggregates multiple pairwise decisions based on the majority voting rule, whereas multi-category D-learning measures the effect of each treatment based on the sum of pairwise contrasts and then picks the treatment with the largest effect measure.

### 4.4 Bayesian additive regression trees (indirect method)

Bayesian additive regression trees (BART) is a flexible nonparametric prediction model that was first introduced by Chipman et al.^39^ and has gained much popularity with numerous applications in recent years.^40,41^ The BART model is an additive ensemble of many single regression trees with each tree explaining a small portion of the outcome, and it can accommodate nonlinear main effects as well as complex interaction effects without the need to specify their functional forms. We refer readers to Tan and Roy^40^ and Hill et al.^41^ for a walk-through of the details on BART.

Logan et al.^10^ incorporated the idea of BART into the estimation of the optimal ITR by using BART to model the dependency structure of the response and covariates. In the first stage, a BART model for E(Y|X,A) was built using the treatment and all covariates as input variables. In the second stage, the optimal arm was chosen as the one that maximizes E(Y|X=x,A=a) , which can be approximated by the average over posterior draws. Under this framework, the posterior distribution of the value function of an ITR can be obtained using posterior samples straightforwardly, and uncertainties about the value of an ITR can be captured by posterior samples from the prediction model directly.

One potential issue of this method is that the prediction model constructed with BART is “black-box”, which impairs the interpretability of the model and the decision rules. In the two-arm setting, Logan et al.^10^ proposed a way to facilitate the interpretation by fitting the posterior mean differences into a tree structure. However, the extension to the multi-arm case is not straightforward given that there are multiple pairwise contrasts when more than two treatment options are available. In addition, BART is more computationally demanding than the aforementioned non-Bayesian approaches due to its reliance on Markov chain Monte Carlo (MCMC).

## 5 Simulation studies

To our knowledge, there is a lack of studies comparing and reporting the performance of different multi-arm ITR estimation methods under scenarios where the training sample size is larger than 2000. It is reasonable to expect that the relative performance of different methods may vary with the training sample size, and thus findings in previous works may not apply to the INTERVAL trial, where the number of male/female participants is more than 20,000. In addition, the BART ITR estimation method^10^ discussed in Section 4.4 has not been compared with other methods in multi-arm trial settings before. In this section, we conduct simulation studies to compare the performance of the following five methods in large samples

- *l*_1_-PLS-HGL: *l*_1_-penalized least squares with hierarchical group LASSO variable selection using the basis function (1,X,A,XA) .^7,32^
- *l*_1_-PLS-GL: *l*_1_-penalized least squares with group LASSO variable selection using the basis function (1,X,A,XA) .^7,30^
- ACWL: Adaptive contrast weighted learning using equation (7), where suboptimal decisions lead to maximal expected loss in the outcome (i.e. ACWL-C2).^8^ We only present the results from ACWL-C2, since we observe that adaptive contrast weighted learning using equation (6) (i.e. ACWL-C1) gives very similar results in simulation studies, as has also been noted in the original publication.^8^
- D-learning: Direct learning with linear decision functions.^9^
- BART: Bayesian additive regression trees with default prior parameters as specified in the R package BART.^10,39^

We consider the training sample size being *n *=* *20,000 (similar in size to the number of male/female donors in the INTERVAL trial). We simulate data under six settings with different types of covariates and forms of treatment–covariate interaction effects. In each simulation setting, we generate five covariates $X_{1},\ldots ,X_{5}$ independently. Treatment *A* is sampled uniformly from {1, 2, 3}. We assume the outcome *Y* is normally distributed with mean m(X)+Δ(X,A) and variance $\sigma^{2}=1$ , where m(X) is the main effect of covariates on the outcome and Δ(X,A) denotes the treatment–covariate interaction effect. We consider $m(X)=1+0.5X_{4}+0.3X_{5}$ and $\Delta \left(X,A\right)=0.5\left\{I(A=1)\Delta_{1}(X)+I(A=2)\Delta_{2}(X)+I(A=3)\Delta_{3}(X)\right\},$ with $\Delta_{1}(X),\;\Delta_{2}(X)$ , and $\Delta_{3}(X)$ taking on different functional forms. Details on distributions of $X_{1},\ldots ,X_{5}$ (we use U{a,b} to denote the continuous uniform distribution that takes values in the range [a,b] , and Bern(*p*) to denote the Bernoulli distribution with the success probability being *p*) and expressions for $\Delta_{1}(X),\;\Delta_{2}(X)$ , and $\Delta_{3}(X)$ in settings 1–6 are provided in Table 1. Settings 1 and 2 consider tree-type and linear interaction effects, respectively. True underlying decision boundaries are nonlinear in settings 3 and 4, with setting 3 including a between-covariate interaction. Setting 5 contains discrete covariates (one categorical covariate and one binary covariate). We examine the scenario where the true optimal treatment is the same for all individuals in setting 6 (this scenario mimics the situation in the INTERVAL trial when we target two outcomes separately, i.e. objectives (1) and (2) described in Section 1: in this case, we would expect the donor assignment strategy to be quite “polar” and the non-personalized rule to yield nearly optimal outcomes based on the primary analysis results presented in Di Angelantonio et al.^12^). We consider additional simulation settings with correlated (Appendix B.1) and higher-dimensional (Appendix B.2) covariates in the online supplement.

**Table 1.** Description of simulation settings 1–6 and simulation results for *n *=* *20,000 based on 100 replicates: mean (SD) of misclassification rates and value functions.

Setting	Functional form of interaction	True optimal treatment	Covariates type	Method	Misclassification	Value
1	Tree $\Delta_{1}\left(X\right)=4\times I\left(X_{1}>0.5\right)-2$ $\Delta_{2}\left(X\right)=2\times I\left(X_{2}\geq 0.5\right)I\left(X_{3}<0.25\right)-1$ $\Delta_{3}(X)=0$	1 or 2 or 3	Continuous $X_{1},\ldots ,X_{5}∼U\{-1,1\}$	*l*_1_-PLS-HGL	0.101 (0.016)	1.226 (0.010)
				*l*_1_-PLS-GL	0.094 (0.015)	1.229 (0.010)
				ACWL	0.028 (0.040)	1.276 (0.023)
				D-learning	0.100 (0.020)	1.226 (0.013)
				BART	**0.010 (0.004)**	**1.286 (0.003)**
2	Linear $\Delta_{1}(X)=3X_{1}-2X_{2}$ $\Delta_{2}(X)=5X_{3}-X_{4}+X_{5}-1$ $\Delta_{3}(X)=0$	1 or 2 or 3	Continuous $X_{1},\ldots ,X_{5}∼U\{-1,1\}$	*l*_1_-PLS-HGL	0.015 (0.004)	1.736 (0.003)
				*l*_1_-PLS-GL	**0.013 (0.004)**	**1.737 (0.003)**
				ACWL	0.171 (0.020)	1.662 (0.016)
				D-learning	0.018 (0.005)	**1.737 (0.004)**
				BART	0.056 (0.004)	1.730 (0.006)
3	Nonlinear $\Delta_{1}(X)=3X_{1}^{2}-\text{exp}(X_{2})$ $\Delta_{2}(X)=X_{3}X_{4}$ $\Delta_{3}(X)=0$	1 or 2 or 3	Continuous $X_{1},\ldots ,X_{5}∼U\{-1,1\}$	*l*_1_-PLS-HGL	0.566 (0.012)	1.089 (0.008)
				*l*_1_-PLS-GL	0.565 (0.014)	1.088 (0.011)
				ACWL	0.561 (0.016)	1.089 (0.009)
				D-learning	0.572 (0.013)	1.087 (0.008)
				BART	**0.192 (0.038)**	**1.209 (0.010)**
4	Nonlinear $\Delta_{1}(X)=3X_{1}^{2}-\text{exp}(X_{2})$ $\Delta_{2}(X)=X_{3}^{3}$ $\Delta_{3}(X)=0$	1 or 2 or 3	Continuous $X_{1},\ldots ,X_{5}∼U\{-1,1\}$	*l*_1_-PLS-HGL	0.350 (0.011)	1.129 (0.004)
				*l*_1_-PLS-GL	0.352 (0.010)	1.128 (0.004)
				ACWL	0.362 (0.011)	1.118 (0.004)
				D-learning	0.359 (0.016)	1.129 (0.004)
				BART	**0.163 (0.045)**	**1.220 (0.012)**
5	Nonlinear $\Delta_{1}(X)=2\{I(X_{1}=1)+I(X_{1}=2)\}X_{2}-1$ $\Delta_{2}(X)=5I(X_{1}=5)X_{3}-2$ $\Delta_{3}(X)=0$	1 or 2 or 3	Continuous + binary+ categorical $X_{1}∼$ discrete uniform {1, 5} $X_{2}∼\text{Bern}(0.5)$ $X_{3},X_{4},X_{5}∼U\{-1,1\}$	*l*_1_-PLS-HGL	0.077 (0.019)	1.101 (0.012)
				*l*_1_-PLS-GL	0.078 (0.018)	1.101 (0.011)
				ACWL	0.029 (0.028)	1.129 (0.016)
				D-learning	0.090 (0.032)	1.094 (0.019)
				BART	**0.007 (0.005)**	**1.142 (0.002)**
6	Tree $\Delta_{1}\left(X\right)=I\left(X_{1}>0.5\right)+2$ $\Delta_{2}\left(X\right)=2\times I\left(X_{2}\geq 0.5\right)\;I\left(X_{3}<0.25\right)-3$ $\Delta_{3}(X)=0$	1	Continuous $X_{1},\ldots ,X_{5}∼U\{-1,1\}$	*l*_1_-PLS-HGL	**0.000 (0.000)**	**2.093 (0.000)**
				*l*_1_-PLS-GL	**0.000 (0.000)**	**2.093 (0.000)**
				ACWL	**0.000 (0.000)**	**2.093 (0.000)**
				D-learning	**0.000 (0.000)**	**2.093 (0.000)**
				BART	**0.000 (0.000)**	**2.093 (0.000)**

Note: Methods under comparison include the *l*_1_-penalized least squares with hierarchical group LASSO variable selection (*l*_1_-PLS-HGL), *l*_1_-penalized least squares with group LASSO variable selection (*l*_1_-PLS-GL), adaptive contrast weighted learning (ACWL), direct learning (D-learning), and Bayesian additive regression trees (BART). The smallest misclassification rates and the largest value functions for each setting are in bold.

We evaluate the performance of different methods on a large independent testing dataset of size 10,000 using two criteria: (a) the misclassification rate of the estimated optimal ITR compared to the true optimal ITR, and (b) the value function under the estimated ITR, which can be calculated according to equation (1). Smaller misclassification rates and larger value functions indicate better performance. Each simulation is repeated 100 times and all tuning parameters are selected via five-fold cross-validation. We report both the mean and the standard deviation of misclassification rates and value functions across 100 replicates. We note that the BART ITR estimation method is different from the other four non-Bayesian methods by nature, and the uncertainty of the value function can be directly quantified under the Bayesian framework (based on posterior samples), as has been discussed in Section 4.4. However, to make different methods comparable, the standard deviation estimates of value functions associated with the BART ITR are calculated as the standard deviation across 100 runs rather than the posterior standard deviation.

Simulation results are presented in Table 1. As expected, when the true underlying decision boundaries are tree-type (setting 1), ACWL that builds on decision trees performs better than *l*_1_-PLS-HGL, *l*_1_-PLS-GL, and D-learning. BART leads to slightly smaller misclassification rate than ACWL in this setting. In contrast, when decision boundaries are linear (setting 2), *l*_1_-PLS-HGL, *l*_1_-PLS-GL, and D-learning perform similarly well and much better than ACWL. BART is superior to ACWL but slightly worse than the other three methods in this case, possibly due to its “over-parameterization” for linear effects. None of these methods manage to capture the nonlinear structures in settings 3 and 4 properly, and misclassification rates are not as low as in other settings for all methods despite the large training sample size. However, BART outperforms the rest of the methods to a large extent. This is not surprising given the flexibility of BART. All methods achieve good performance when some covariates are discrete (setting 5). Results for setting 6 imply that when the true optimal treatment is the same for all subjects (“trivial” decision rule that assigns all to the marginally best treatment), all methods perform perfectly with no misclassification. We also conduct simulations for some variations of setting 6 (e.g. increased noise) and find similar results (online supplement Appendix B.3).

Overall, our simulation studies suggest that unless we have *a priori* information on the type of underlying interaction effects (e.g. linear additive or tree-type), the BART multi-arm ITR estimation method should be a good choice in general since it is robust and performs better than or comparable to the other competing methods regardless of the functional form of interaction terms. We note that BART takes longer to run than other non-Bayesian methods, especially in the large sample size case that we examine in this paper (on a Windows-based computing system with 1 core, 3.40 GHz Intel processor, BART takes about 200 seconds per run while other methods take less than 20 seconds per run on our simulated datasets with 20,000 observations). Parallel processing with multi-threading can be used to speed up the computation of BART.^42^ We also note that in settings with nonlinear decision boundaries, the performance of *l*_1_-PLS and D-learning may be improved if we use polynomials of higher degrees as basis functions. For D-learning, we would also expect the nonlinear version which estimates the optimal decision function using the component selection and smoothing operator (COSSO) to perform better than the linear version considered in this paper if the underlying decision boundaries are nonlinear. However, our numerical experiments suggest that nonlinear D-learning with COSSO is much more computationally demanding and performs less well than BART when *n *=* *20,000.

R codes to implement these methods on a simulated example dataset are provided at https://github.com/yux139/ITR_multiarm_casestudy.

## 6 Application to the INTERVAL trial

To illustrate the use of multi-arm ITR estimation methods on large-scale clinical trials, we apply the five methods compared in Section 5 to the data from male donors in the INTERVAL trial and estimate each blood donor’s optimal inter-donation interval. We also compare the personalized donation strategies with “one-size-fits-all” donor assignment rules that recommend the same inter-donation intervals of 8, 10 or 12 weeks for all male donors. After data processing (exclude donors who had zero attendance over the two-year trial period), 20,574 male blood donors are included in the analysis following the intention-to-treat principle according to donors’ randomized groups. We show in the online supplemental material (Appendix C) that the data cleaning process does not distort the balance of baseline covariates across randomized groups.

### 6.1 Outcomes of interest

We consider two outcomes, namely, the total units of blood collected by the blood service per donor over a two-year period (the standard practice is to donate 1 unit of blood per session, with a full donation unit containing 470 ml of whole blood^43^), denoted by *G*, and the rate of low Hb deferrals per donor attendances during the same period (calculated as the total number of “at session” deferrals for low Hb divided by the total number of attendances in the two-year trial period), denoted by *R*. We note that these two outcomes are not independent: assigning a donor to a more frequent inter-donation interval in principle will lead to an increase in the total units of blood collected. However, this increased frequency may have the opposite effect through increased risks of deferrals for low Hb, which may consequently cause existing donors to come back less often and even to leave the donor register permanently.^11,12,44^ Potential loss of donors may have a cost impact.^44,45^ The current donor loss rate following a deferral for low Hb is 40–50%, and this would incur substantial costs (approximately £2.3 million in the worst-case scenario) for the blood service to recruit sufficient new donors and stabilize the donor base.^46,47^ Therefore, when recommending the optimal inter-donation interval to a blood donor, there is a trade-off between the benefit and the risk: neither the optimal ITR solely based on the benefit nor that solely based on the risk may be acceptable, and it is generally not possible to find a strategy that optimizes both (maximizes benefit and minimizes risk) simultaneously. The goal of maximizing the total units of blood collected needs to be considered in conjunction with controlling for the low Hb deferrals. This motivates us to construct a single scalar “utility” outcome which *discounts* the units of blood collected by the increased incidences of low Hb deferrals as follows

(10) $$\begin{array}{l} U=G-b\times \tilde{R} \end{array}$$

where *G* is the gain/benefit (total units of blood collected in the two-year trial period), $\tilde{R}$ is the risk (number of deferrals for low Hb in the two-year trial period: $\tilde{R}$ = R × total number of attendances over two years), and *b* is the “trade-off” parameter reflecting the equivalent benefit loss for one unit increase in the risk. In the context of the INTERVAL trial, *b* is interpreted as “the equivalent loss in total units of blood collected by the blood service per donor over two years for one extra deferral for low Hb per donor attendances during the same period”. We examine different values for *b* within a range considered to be reasonable by NHSBT and vary *b* from 1 to 5 at an increment of 1 to see how results change with this parameter. A range of *b* from 1 to 5 covers the range of the extra costs of deferrals for low Hb considering reduced efficiency of collection, reduced donor retention and increase in recruitment of the many new donors to replace a regular donor who retires from donation.

We assume a larger outcome to be more desirable when we introduce the statistical framework in Section 3.1. This holds for the benefit and the utility outcome, but is not the case for the low Hb deferral rates. We address this issue by considering the maximization of “1–low Hb deferral rates” instead, which is equivalent to the minimization of the low Hb deferral rates. In addition, the low Hb deferral rates is a proportion and so we use the arcsine square root transformation for variance stabilization.^48^ This transformation is monotonically increasing, and thus rank-preserving.

### 6.2 Baseline covariates

Based on findings in the primary trial paper,^12^ we include the following 19 variables measured at each donor’s baseline visit:

- Continuous: age, body mass index, Short Form Health Survey version 2 (SF-36v2) physical component score and mental component score, units of whole blood donations in the two years before enrollment into the trial, hemoglobin level, white blood cell count, red blood cell count, mean corpuscular hemoglobin, mean corpuscular volume, and platelet count.
- Categorical: ethnicity (Asian, Black, Mixed, White, Other, Unknown), blood group (A+, A−, AB+, AB−, B+, B−, O+, O−), iron prescription (Yes, No, Unknown), smoke ever (Yes, No, Unknown), smoke currently (Yes, No, Unknown), alcohol ever (Yes, No, Unknown), alcohol currently (Yes, No, Unknown), and new or returning donor status (New donor, Returning donor).

### 6.3 Evaluation criteria

We calculate proportions of donors assigned to each of the three inter-donation intervals according to the optimal ITR estimated using different methods. We are also interested in the quantity “ITR effect” or “benefit function”.^49,50^ The ITR effect, *δ*, associated with the rule D(X) is defined as

(11) $$\begin{array}{l} \delta \left(D\left(X\right)\right)=E\left\{Y|X,A=D(X)\right\}-E\left\{Y|X,A\neq D(X)\right\} \end{array}$$

In the INTERVAL trial, the ITR effect can be empirically estimated as the difference in the average outcome between donors whose assigned inter-donation intervals in the trial are the same as D(X) (i.e. average across all donors whose a=D(x) ) and those whose assigned inter-donation intervals are different from D(X) (i.e. average across all donors whose a≠D(x) ).

When two outcomes are analyzed separately, in addition to ITR effects of the estimated optimal assignment rule $\hat{D^{*}}(X)$ on the outcome that we aim to optimize, we also calculate the effect of assigning donors according to $\hat{D^{*}}(X)$ on the other outcome that we do not take into consideration when estimating $D^{*}(X)$ .

When we consider the combined outcome and aim at maximizing the utility score, ITR effects on the units of blood collected, *G*, the low Hb deferral rates, *R*, and the utility outcome, *U*, are computed by replacing *Y* in equation (11) with *G*, *R*, and *U*, respectively. A larger ITR effect on donation and utility, and a smaller ITR effect on deferral are more desirable.

### 6.4 Non-Bayesian approaches: l_1_-PLS, ACWL, and D-learning

We first apply the *l*_1_-PLS with HGL/GL variable selection, ACWL, and D-learning to data from male donors in the INTERVAL trial. In each of the analyses, we randomly split the data into a training and a validation set with a 4:1 ratio and repeat the procedure 100 times. All tuning parameters are selected via five-fold cross-validation. For each method, we report the means and the standard deviations of empirical assignment proportions and ITR effects evaluated on the validation data across 100 splits. Note that the standard deviation estimates based on cross-validation capture the randomness of data-splitting (“repeatability”) rather than the uncertainty of the observed data. We provide bootstrap-based standard deviation estimates that reflect the “biological variation” in the online supplemental materials (Appendix D).

#### 6.4.1 Target two outcomes separately

Table 2 presents the results for analyzing the donation and deferral outcomes separately. As expected from the simulation results, we observe a consistent pattern across different methods in these extreme cases where true optimal decisions should be “almost trivial” and the non-personalized strategy that assigns everyone to the marginally best “treatment” should lead to almost optimal outcomes. As suggested by the “assignment percentages” columns in Table 2, almost all donors (ranging from 99.4% to 100.0%) are assigned to the shortest inter-donation interval (eight-week) if the goal is to maximize the total units of blood collected by the blood service, and ITRs estimated using these precision medicine-based methods are indeed very close to “one-size-fits-all” rules. In contrast, if our aim is to minimize the low Hb deferral rates, then the longest inter-donation interval (12-week) should be recommended for most donors (ranging from 94.4% to 99.7%). These findings are consistent with results from the primary analysis of the INTERVAL trial data.^12^

**Table 2.** Applications of the *l*_1_-penalized least squares with hierarchical group LASSO variable selection (*l*_1_-PLS-HGL), *l*_1_-penalized least squares with group LASSO variable selection (*l*_1_-PLS-GL), adaptive contrast weighted learning (ACWL), and direct learning (D-learning) to data from male donors in the INTERVAL trial.

		Assignment Percentages			ITR Effects	
Target Outcome	Method	12 weeks	10 weeks	8 weeks	Donation	Deferral
Donation	*l*_1_-PLS-HGL	0.1 (0.0)	0.3 (0.0)	99.6 (0.0)	1.308 (0.004)	0.026 (0.000)
	*l*_1_-PLS-GL	0.0 (0.0)	0.3 (0.3)	99.7 (0.3)	1.311 (0.005)	0.027 (0.000)
	ACWL	0.0 (0.0)	0.0 (0.0)	100.0 (0.0)	1.315 (0.000)	0.027 (0.000)
	D-learning	0.3 (0.1)	0.3 (0.2)	99.4 (0.2)	1.307 (0.006)	0.027 (0.000)
Deferral	*l*_1_-PLS-HGL	94.4 (0.6)	5.5 (0.6)	0.0 (0.0)	−1.188 (0.010)	−0.024 (0.000)
	*l*_1_-PLS-GL	99.7 (0.6)	0.2 (0.5)	0.0 (0.1)	−1.246 (0.006)	−0.024 (0.000)
	ACWL	99.7 (0.6)	0.3 (0.6)	0.0 (0.0)	−1.244 (0.007)	−0.025 (0.000)
	D-learning	95.7 (0.5)	4.1 (0.5)	0.2 (0.1)	−1.200 (0.011)	−0.024 (0.000)

Note: Means and standard deviations (in parenthesis) of assignment proportions in % and empirical ITR effects on donation and deferral outcomes across 100 repetitions of 5-fold cross-validation are reported. ITR effects measure the difference in the average outcome between donors whose assigned inter-donation intervals in the trial are optimal (with respect to the method used to estimate the ITR) and those whose assigned inter-donation intervals are non-optimal. A larger ITR effect on donation and a smaller ITR effect on deferral are more desirable. The first four and last four rows correspond to the target being maximizing total units of blood collected by the blood service, and minimizing the low Hb deferral rates, respectively.

For comparison, we also calculate the ITR effects on donation and deferral of three non-personalized rules where the same inter-donation interval is recommended for all donors. ITR effects of non-personalized (fixed) rules measure the difference in the average outcome between donors whose assigned inter-donation intervals in the trial are the same as the one specified in the fixed rule and those whose assigned inter-donation intervals are different from that specified in the fixed rule. We still use equation (11) to calculate ITR effects of fixed rules except that D(X) is replaced with fixed rules that do not depend on **X**. ITR effects on donation are −1.248, −0.077, and 1.315 for assigning all donors to the 12-, 10-, and eight-week inter-donation interval, respectively; and ITR effects on deferral are −0.025, −0.002, and 0.027 for assigning all donors to the 12-, 10-, and eight-week inter-donation interval, respectively. This suggests that if the sole interest is in collecting more blood, the non-personalized rule that recommends all donors to donate every eight weeks leads to the largest increase in the units of blood collected compared to personalized rules estimated using different methods. On the other hand, if we are only concerned with minimizing deferrals for low Hb, then the non-personalized rule that recommends all donors to the 12-week inter-donation interval yields the largest reduction in the rate of low Hb deferrals compared to personalized rules. However, we also observe from Table 2 that by following the optimal rule for maximizing the total units of blood collected by the blood service, the average increase in blood donations is about 1.31 units (616 ml) per donor over two years, but at the same time, there is also an increasing number of deferrals for low Hb at about 2.7 per 100 donor attendances on average. This is consistent with our intuition: an “optimal” rule that maximizes clinical benefits also leads to safety concerns (high risks of adverse events), and vice versa. Therefore, it is necessary to strike a balance between the two “competing” outcomes by maximizing the utility score which incorporates the trade-off between the benefit and the risk.

#### 6.4.2 Target the utility outcome

**Figure 1.**
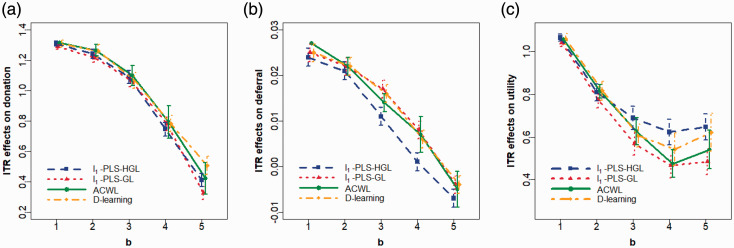
Plots of the mean and 95% confidence intervals for ITR effects of the optimal ITR estimated using various methods as the trade-off parameter *b* in the utility function varies from 1 to 5 at an increment of 1. Optimal ITRs are estimated using data from male donors in the INTERVAL trial assuming the target is to maximize the utility. Methods to estimate the optimal ITR include *l*_1_-penalized least squares with hierarchical group LASSO variable selection (*l*_1_-PLS-HGL), *l*_1_-penalized least squares with group LASSO variable selection (*l*_1_-PLS-GL), adaptive contrast weighted learning (ACWL), and direct learning (D-learning). ITR effects on the (a) donation, (b) deferral, and (c) utility outcomes are presented. A larger ITR effect on donation/utility and a smaller ITR effect on deferral are more desirable.

Table 3 summarizes allocation proportions and ITR effects associated with $\hat{D^{*}}(X)$ for different values of the trade-off parameter *b* in the utility function, and Figures 1(a) to (c) plot the relationships between estimated ITR effects (on donation, deferral and utility outcomes, respectively) and *b* when the target is to maximize the utility score. Again, we observe very similar results using different methods (“similar” in terms of clinical meaningfulness), especially when the value of *b* is small. As *b* increases, assignment proportions shift from the more frequent to less frequent inter-donation intervals: less donors are allocated to the shortest inter-donation interval, and more donors are allocated to the longest one. Consequently, both the increase in the total units of blood collected (i.e. benefit) and the increase in the low Hb deferral rates (i.e. risk) become smaller. When *b* takes the value from 1 to 4, both benefit and risk increase if the target is to maximize corresponding utilities, while in the extreme case where *b *=* *5, there is an increase in the benefit and a decrease in the risk. As a reference, we also calculate the ITR effects of three non-personalized rules on the utility outcome when the trade-off parameter *b* varies from 1 to 5 (Table 4) and compare with those of personalized rules presented in Table 3. Unlike the case when we analyze two outcomes separately where the “one-size-fits-all” rule seems to be sufficient for achieving a desirable outcome, when we target the utility outcome, personalized rules that tailor to each donor’s capacity to donate lead to a higher gain in utility scores compared to non-personalized rules, and the advantage over non-personalized rules becomes more pronounced as *b* increases. For example, when *b *=* *5, ITR effects on the utility score of the best non-personalized rule (assign all donors to the 10-week inter-donation interval) is 0.183, while ITR effects of personalized rules range from 0.485 to 0.648, depending on which method is used to estimate the optimal ITR.

**Table 3.** Applications of the *l*_1_-penalized least squares with hierarchical group LASSO variable selection (*l*_1_-PLS-HGL), *l*_1_-penalized least squares with group LASSO variable selection (*l*_1_-PLS-GL), adaptive contrast weighted learning (ACWL), and direct learning (D-learning) to data from male donors in the INTERVAL trial assuming the target is to maximize the utility. The trade-off parameter b in the utility function varies from 1 to 5 at an increment of 1.

		Assignment Percentages			ITR Effects		
Trade-off Parameter	Method	12 weeks	10 weeks	8 weeks	Donation	Deferral	Utility
*b* = 1	*l*_1_-PLS-HGL	0.9 (0.1)	1.2 (0.1)	97.9 (0.1)	1.309 (0.008)	0.024 (0.001)	1.064 (0.009)
	*l*_1_-PLS-GL	0.3 (0.1)	2.4 (0.8)	97.2 (1.0)	1.289 (0.014)	0.025 (0.001)	1.040 (0.014)
	ACWL	0.0 (0.0)	0.0 (0.1)	100.0 (0.1)	1.314 (0.003)	0.027 (0.000)	1.055 (0.002)
	D-learning	0.7 (0.2)	1.2 (0.5)	98.1 (0.5)	1.309 (0.012)	0.025 (0.001)	1.058 (0.013)
*b* = 2	*l*_1_-PLS-HGL	3.4 (0.1)	4.2 (0.3)	92.4 (0.4)	1.242 (0.016)	0.021 (0.001)	0.809 (0.020)
	*l*_1_-PLS-GL	1.7 (0.4)	7.2 (1.6)	91.1 (2.0)	1.217 (0.027)	0.022 (0.002)	0.774 (0.024)
	ACWL	2.7 (0.8)	3.4 (1.7)	93.9 (1.3)	1.266 (0.019)	0.022 (0.001)	0.814 (0.016)
	D-learning	1.5 (0.4)	5.8 (1.1)	92.7 (1.0)	1.260 (0.022)	0.022 (0.001)	0.816 (0.023)
*b* = 3	*l*_1_-PLS-HGL	8.6 (0.3)	11.9 (0.5)	79.5 (0.6)	1.091 (0.022)	0.011 (0.001)	0.689 (0.028)
	*l*_1_-PLS-GL	4.8 (1.1)	15.0 (3.3)	80.2 (4.4)	1.069 (0.056)	0.017 (0.003)	0.569 (0.041)
	ACWL	9.3 (1.4)	8.2 (2.7)	82.5 (2.2)	1.100 (0.034)	0.014 (0.001)	0.627 (0.032)
	D-learning	3.8 (0.8)	17.5 (1.6)	78.6 (1.3)	1.067 (0.027)	0.016 (0.001)	0.607 (0.027)
*b* = 4	*l*_1_-PLS-HGL	17.0 (0.4)	23.3 (0.5)	59.7 (0.5)	0.745 (0.023)	0.001 (0.001)	0.623 (0.030)
	*l*_1_-PLS-GL	10.5 (2.3)	27.9 (3.2)	61.6 (5.2)	0.782 (0.070)	0.008 (0.004)	0.468 (0.081)
	ACWL	16.8 (1.9)	16.2 (3.5)	67.0 (3.1)	0.793 (0.055)	0.007 (0.002)	0.475 (0.033)
	D-learning	9.9 (1.6)	30.0 (1.7)	60.2 (0.7)	0.783 (0.027)	0.006 (0.001)	0.543 (0.039)
*b* = 5	*l*_1_-PLS-HGL	26.4 (0.4)	33.4 (0.5)	40.3 (0.3)	0.410 (0.022)	−0.007 (0.001)	0.648 (0.031)
	*l*_1_-PLS-GL	18.2 (3.2)	48.4 (6.2)	33.4 (3.4)	0.324 (0.059)	−0.004 (0.002)	0.485 (0.084)
	ACWL	30.1 (2.5)	22.6 (4.2)	47.3 (3.2)	0.422 (0.053)	−0.005 (0.002)	0.541 (0.046)
	D-learning	19.3 (1.6)	37.4 (1.3)	43.3 (0.7)	0.505 (0.031)	−0.004 (0.001)	0.622 (0.045)

Note: Means and standard deviations (in parenthesis) of assignment proportions in % and empirical ITR effects on donation, deferral, and utility across 100 repetitions of 5-fold cross-validation are reported. ITR effects measure the difference in the average outcome between donors whose assigned inter-donation intervals in the trial are optimal (with respect to the method used to estimate the ITR) and those whose assigned inter-donation intervals are non-optimal. A larger ITR effect on donation/utility and a smaller ITR effect on deferral are more desirable.

**Table 4.** ITR effects of three non-personalized rules on the utility outcome. The trade-off parameter b in the utility function varies from 1 to 5 at an increment of 1.

	ITR Effects on Utility				
Non-personalized Rule	*b* = 1	*b* = 2	*b* = 3	*b* = 4	*b* = 5
Recommend all male donors to donate every 12 weeks	−1.308	−0.828	−0.618	−0.408	−0.199
Recommend all male donors to donate every 10 weeks	−0.025	0.027	0.079	0.131	0.183
Recommend all male donors to donate every 8 weeks	1.055	0.795	0.535	0.275	0.015

Note: ITR effects measure the difference in the average outcome between donors whose assigned inter-donation intervals in the trial are the same as the one specified in the non-personalized rule and those whose assigned inter-donation intervals are different from that specified in the non-personalized rule. A larger ITR effect on utility is more desirable.

#### 6.4.3 Variable selection by l_1_-PLS-HGL and l_1_-PLS-GL

In addition to estimating the optimal ITR, *l*_1_-PLS with group LASSO (GL) or hierarchical group LASSO (HGL) variable selection also picks important treatment–covariate interactions when building the prediction model for E(Y|X,A) . Despite the mismatch issue discussed in Section 4.1, we are interested in investigating which treatment–covariate interactions are estimated as non-zero and regarded as important in the prediction model. For demonstration, we focus on the situation where maximizing the total units of blood collected by the blood service is our primary goal. Table 5 summarizes selection percentages (across 100 repetitions of five-fold cross-validation) of different treatment–covariate interactions in the prediction model for donation when group LASSO that does not impose strong hierarchy between main effects and interactions, or hierarchical group LASSO that enforces such hierarchy is used for variable selection.

**Table 5.** Selection percentages of treatment-covariate interactions in the prediction model for the donation outcome across 100 repetitions of 5-fold cross-validation when *l*_1_-PLS is used to estimate the optimal ITR.

		Variable Selection	
Baseline Variables	Variable Type	HGL	GL
Age	Continuous	63	21
Body mass index	Continuous	33	38
SF-36v2 physical component score	Continuous	47	11
SF-36v2 mental component score	Continuous	100	20
Blood donations in the two years before trial enrollment	Continuous	100	100
Hemoglobin level	Continuous	100	100
White blood cell count	Continuous	100	46
Red blood cell count	Continuous	0	11
Mean corpuscular hemoglobin	Continuous	100	96
Mean corpuscular volume	Continuous	62	11
Platelet count	Continuous	100	12
Ethnicity	Categorical	47	97
Blood group	Categorical	99	99
Iron prescription	Categorical	1	74
Smoke ever	Categorical	13	39
Smoke currently	Categorical	98	99
Alcohol ever	Categorical	85	32
Alcohol currently	Categorical	100	100
New or returning donor status	Categorical	100	32

Note: Hierarchical group LASSO (HGL) enforces strong hierarchy between main effects and interactions, and group LASSO (GL) does not impose strong hierarchy between main effects and interactions.

We observe that some interactions between the randomized group (inter-donation interval) and baseline characteristics are selected almost all the time by both variable selection methods, such as blood donations in the two years before trial enrollment, baseline hemoglobin level, blood group, etc. We also notice that for some baseline covariates, selection percentages of their interaction with the randomized group differ substantially between the two variable selection approaches, even though the donor assignment proportions and ITR effects estimated using *l*_1_-PLS-HGL and *l*_1_-PLS-GL (Table 2) are very similar. A possible explanation for an interaction being selected much more often by HGL than by GL (the case for most continuous covariates) is that the effect of the interaction itself is not strong enough and may be dominated by the main effect, but as has been noted in Lim and Hastie,^33^ their proposed HGL method can still discover important interaction terms in this case due to the use of the “glinternet” penalty.

It is worth noting that the high selection rates of interactions between randomized groups and baseline covariates do not imply different recommendations on the inter-donation interval for different donors, and the finding that almost all male donors should donate every eight weeks to maximize the total units of blood collected (Table 2) does not contradict the observation that many interaction effects are estimated to be non-zero (Table 5). This is because variable selection methods that we use to identify important interactions do not distinguish between quantitative and qualitative interactions, whereas only qualitative interactions can lead to different inter-donation interval recommendations for different subpopulations. In this dataset, it might be the case that even though interactions exist, they are mostly quantitative, as has also been observed in the pre-specified subgroup analysis in the INTERVAL trial.^12^

### 6.5 The Bayesian approach: BART

Following Logan et al.,^10^ we first build the conditional mean outcome model using BART, and then we identify the optimal ITR based on posterior predictive distributions of the conditional mean under each randomized group (the resulting optimal ITR is referred to as the “BART ITR”). According to Section 4.4, the BART ITR is the one in which the recommended arm for each individual is given by maximizing the subject-specific MCMC estimate (e.g. posterior mean) of the posterior predictive distribution of E(Y|X=x,A=a) . In the INTERVAL trial, the BART ITR is the rule that assigns each donor to the inter-donation interval that leads to the largest posterior mean of utilities/total units of blood collected, or the inter-donation interval that corresponds to the smallest posterior mean of low Hb deferral rates.

#### 6.5.1 Target two outcomes separately

Consistent with our findings using frequentist approaches, when we fit the BART model to the INTERVAL data with the target of maximizing the total units of blood collected by the blood service, the BART ITR, ${\hat{D^{*}}}_{\text{BART}}(X)$ , assigns all male donors to the most frequent inter-donation interval (eight-week) with the posterior mean of the ITR effects on donation being 1.313 (95% credible interval: [1.237,1.392]). On the other hand, when the aim is to minimize low Hb deferral rates, ${\hat{D^{*}}}_{\text{BART}}(X)$ recommends 91.6% of male donors to donate every 12 weeks, leading to an ITR effect of −0.024 (posterior mean) on the deferral outcome with the 95% credible interval being [−0.027, −0.022].

#### 6.5.2 Target the utility outcome

We also fit the BART model to the INTERVAL data with the utility score being the target outcome and obtain the posterior predictive distribution of the utility score under each inter-donation interval option. We vary the trade-off parameter *b* in the utility function from 1 to 5 at an increment of 1 to see how ${\hat{D^{*}}}_{\text{BART}}(X)$ changes with this parameter. For each *b*, we compare five donor assignment rules:

- All donors donate every eight weeks;
- All donors donate every 10 weeks;
- All donors donate every 12 weeks;
- Donors donate according to the BART ITR (each donor is assigned to the inter-donation interval associated with the maximum posterior mean of the utility score);
- Donors donate according to the optimized ITR based on the BART estimation (an idealized scenario in which for each MCMC draw from the posterior distribution, each donor is assigned to the inter-donation interval associated with the maximum value of the utility score. This is non-achievable in practice, but we use this rule as a reference for the best-case scenario).

**Figure 2.**
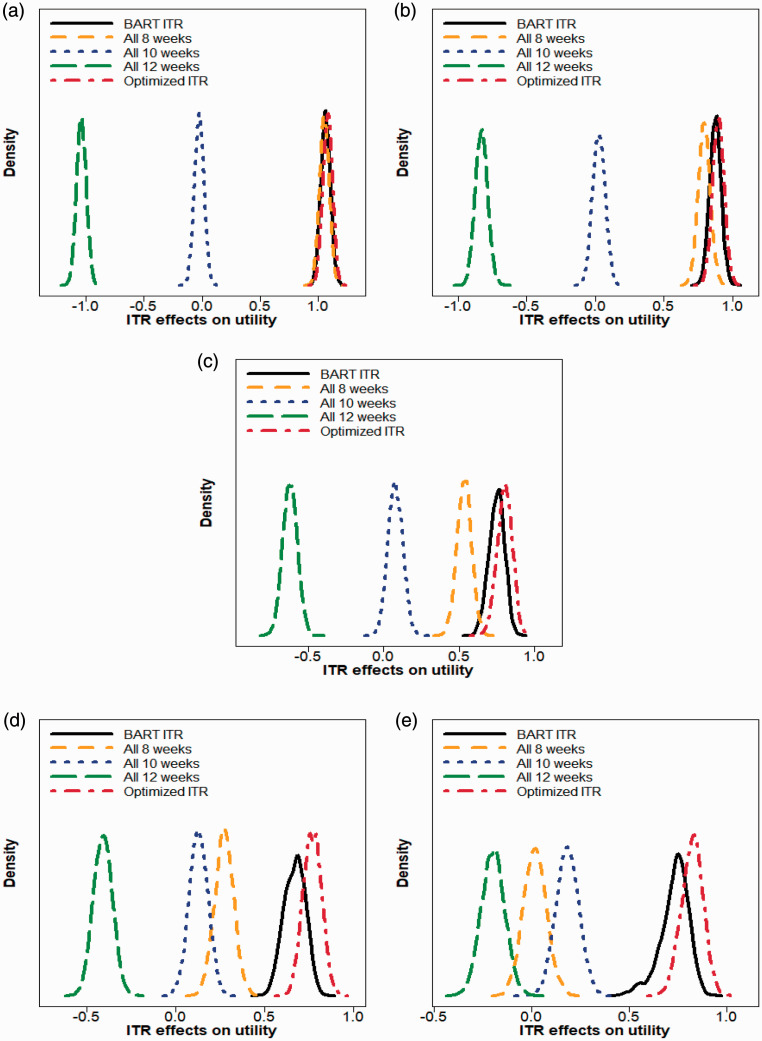
Density plots of ITR effects on utility when (a) *b* = 1 (b) *b* = 2 (c) *b* = 3 (d) *b* = 4 (e) *b* = 5 for five donor assignment rules: recommend all male donors to (i) donate every eight weeks, (ii) donate every 10 weeks, (iii) donate every 12 weeks, (iv) donate according to the BART ITR, and (v) donate according to the optimized ITR (non-achievable in practice). The trade-off parameter *b* in the utility function varies from 1 to 5 at an increment of 1. A larger ITR effect on utility is more desirable.

Posterior distributions of the ITR effects associated with those five donor assignment rules corresponding to five different trade-off parameters are plotted in Figure 2. In Table 6, we summarize donor allocation proportions based on the BART ITR, and we also report the posterior mean and the 95% equal tail credible interval of the ITR effect for each donor assignment strategy (we note that standard deviation estimates reported in Table 3 for non-Bayesian methods are calculated across 100 repetitions of five-fold cross-validation, while in Table 6, we quantify the uncertainty of the estimates directly based on posterior samples from BART).

**Table 6.** Applications of Bayesian additive regression trees (BART) to data from male donors in the INTERVAL trial assuming the target is to maximize the utility score. The trade-off parameter *b* in the utility function varies from 1 to 5 at an increment of 1.

		Trade-off Parameter				
Criteria	Assignment Rule	*b* = 1	*b* = 2	*b* = 3	*b* = 4	*b* = 5
BART ITRAssignment Percentages	12 weeks	0.0	3.6	10.7	17.5	26.7
	10 weeks	3.2	9.4	18.8	25.5	28.4
	8 weeks	96.8	87.1	70.5	57.0	44.9
ITR Effects on UtilityPosterior Mean [95% Credible Interval]	All 12 weeks	−1.037[−1.118,−0.956]	−0.827 [−0.915,−0.739]	−0.618 [−0.712,−0.524]	−0.408 [−0.511,−0.305]	−0.199 [−0.317,−0.082]
	All 10 weeks	−0.025 [−0.106, 0.055]	0.027 [−0.060, 0.114]	0.079 [−0.017, 0.174]	0.131 [0.027, 0.232]	0.183 [0.068, 0.297]
	All 8 weeks	1.054 [0.973, 1.134]	0.794 [0.708, 0.883]	0.535 [0.437, 0.628]	0.275 [0.169, 0.379]	0.016 [−0.101, 0.135]
	BART ITR	1.064 [0.983, 1.144]	0.876 [0.793, 0.959]	0.750 [0.643, 0.843]	0.671 [0.553, 0.780]	0.732 [0.548, 0.852]
	Optimized ITR	1.082 [1.000, 1.159]	0.898 [0.814, 0.981]	0.802 [0.701, 0.893]	0.770 [0.671, 0.870]	0.823 [0.710, 0.928]

Note: Assignment proportions of the BART ITR in percentage and the posterior mean [95% equal tail credible interval] of the ITR effect on the utility outcome for five donor assignment rules are reported. Assignment rules include the following: recommend all male donors to (i) donate every eight weeks, (ii) donate every 10 weeks, (iii) donate every 12 weeks, (iv) donate according to the BART ITR, and (v) donate according to the optimized ITR (non-achievable in practice). A larger ITR effect on utility is more desirable.

We observe that when *b* is small (e.g. *b *=* *1 or 2), the best “one-size-fits-all” strategy is to recommend all donors to donate every 8 weeks. In these cases, both the BART ITR and the optimized ITR are close to the “all 8 weeks” rule in that the ITR effect distribution of the BART ITR and the optimized ITR almost overlaps with that of the “all 8 weeks” rule. When *b* takes values from 3 to 5, the BART ITR is better than the best non-personalized assignment rule (“all 8 weeks” for *b *=* *3, 4, and “all 10 weeks” for *b *=* *5) with a high probability, and the advantage of the BART ITR over non-personalized rules becomes more pronounced as *b* gets larger. Even though the BART ITR is slightly inferior to the optimized ITR (which is generally not achievable in practice) as expected, the differences are minimal.

### 6.6 Measure of agreement between methods

Results presented in Section 6.4 and 6.5 suggest that donor assignment proportions and empirical ITR effects are fairly similar (at the population level) across different methods for each target outcome. We are also interested in investigating for a given male donor, to what extent the five methods (*l*_1_-PLS-HGL, *l*_1_-PLS-GL, ACWL, D-learning, and BART) “agree” on his optimal inter-donation interval (at the individual level). Given the differences in the decision boundary types assumed by each method, we expect that the form of estimated regimes based on different methods may be very different and the set of baseline characteristics included in the optimal decision rule may also vary across methods. However, the optimal inter-donation interval estimated by different approaches for a given donor can still “overlap” despite the possible heterogeneity in the form of estimated regimes.^36^

To evaluate the degree of agreement between different methods in terms of the recommended optimal inter-donation interval for each male donor in the INTERVAL trial, we present two inter-method agreement measures (overall and pairwise). Details and results can be found in the Supplemental materials (Appendix E). We conclude that in most cases, the level of agreement is “substantial” or “almost perfect” based on the values of the pairwise B statistics^51^ and the guidelines for assessing agreement by Munoz and Bangdiwala.^52^

## 7 Discussion

### 7.1 Concluding remarks

Most statistical methods for estimating the optimal individualized treatment rule (ITR) are restricted to binary treatment comparisons. However, clinical studies with more than two treatment arms are common in practice. In this paper, we review several recent approaches that can be used to estimate the optimal ITR in large-scale clinical studies with more than two treatment options. Methods considered include: the *l*_1_-penalized least squares with hierarchical group LASSO or group LASSO variable selection, the adaptive contrast weighted learning method, the direct learning method, and a Bayesian approach that builds on Bayesian additive regression trees.

We conduct simulation studies to evaluate the performance of these methods in large-scale clinical trials. Our simulation results suggest that the BART multi-arm ITR estimation method has better or similar performance compared to other methods across different settings (with different types of interaction terms). In addition, when the sample size is large (as in the INTERVAL trial), all methods perform equally well under scenarios where baseline characteristics interact with treatment arms only quantitatively (and thus the true optimal treatment is the same for all subjects) in the sense that they all accurately identify the true universal decision rules (assign all to the marginally best one).

We further illustrate the use of these methods by applying them to the data collected from male donors in the INTERVAL trial. Results are fairly consistent across different approaches in terms of clinical meaningfulness. When the target is to maximize the total units of blood collected by the blood service, or to minimize the low Hb deferral rates (i.e. the benefit outcome and the risk outcome are not considered in conjunction with each other), all methods detect almost no qualitative heterogeneity of the “inter-donation interval” effects. More specifically, if we focus on maximizing the total units of blood collected, then the optimal ITR tends to choose shorter inter-donation intervals that are associated with increased benefits at the cost of higher risks of deferrals for low Hb and almost all donors are assigned to the highest frequency of donation. On the other hand, if our aim is to minimize the low Hb deferral rates, then the optimal ITR picks longer inter-donation intervals and almost everyone is assigned to the lowest frequency of donation. These results are not surprising and support the trial’s primary findings that interactions between baseline characteristics and the inter-donation interval are not qualitative, but rather quantitative, and almost all donors are able to give blood more frequently than the current standard practice.^12^ Maximizing the benefit and minimizing the risk are two competing goals. While the optimal decision for each goal is obvious, the two decisions may be very different. To deal with this, we create a utility score that balances two outcomes and derive the optimal ITR with the goal of maximizing the utility score. Investigation of the utility outcome suggests some heterogeneity in the optimal inter-donation interval across donors with different baseline characteristics, and such heterogeneity becomes larger as the trade-off parameter *b* in the utility function (the equivalent benefit loss for one unit increase in risk) gets larger.

### 7.2 Generalizability of estimated ITRs

One issue that warrants highlighting is the generalizability of estimated ITRs to a broader population. In general, the entry criteria for clinical trials are restrictive, and trial participants may not be representative of the more general population. Therefore, failure to detect qualitative interactions between baseline characteristics and the treatment assignment among trial participants does not preclude the existence of such qualitative interactions over the entire eligible population, and we should be cautious about generalizing ITRs estimated using trial data to a broader population.^31,53^ However, this may not be a major problem for the INTERVAL trial. As suggested by Moore et al.,^17^ participants in the INTERVAL trial were broadly representative of the national donor population of England, and it is likely that estimated ITRs based on INTERVAL data are generalizable to the general donor population.

We note that the INTERVAL data cannot be used to validate the estimated optimal donation strategies. A confirmatory follow-up trial comparing estimated rules with the current clinical practice should be conducted before applying estimated rules to future donors.

We also comment that the generalizability and validation issues discussed in this section regarding the estimated optimal ITRs are relevant not only to the INTERVAL trial but also more generally to other trials, as have been discussed in literatures.^27,31,53,54^

### 7.3 Model extensions and future work

We can extend this work in several directions. We treat the inter-donation interval as a nominal variable with three categories. However, there is a natural ordering of the 12-, 10-, and eight-week inter-donation interval and these three options can be considered as three levels of an ordinal “treatment”. We would expect to have some information loss by treating an ordinal variable as nominal. Thus, it would be of great interest to explore ways to efficiently incorporate information on the ordinality into decision-making and investigate how much we gain by doing this.

We note that the optimal donor assignment strategy is highly dependent on *b*. We examine different values for *b* within a reasonable range and estimate the optimal ITR under each *b*. Ideally, *b* should be specified based on clinicians’ domain knowledge. However, there is a lack of accurate information on *b* in most cases because the right balance between the benefit and the risk is usually not obvious and can be different for different subgroups.^55^ Kosorok and Laber^2^ summarized other recent work on addressing multiple outcomes in precision medicine. For example, an alternative approach to handle the trade-off between the risk and the benefit is through the constrained optimization framework, i.e. consider maximizing the benefit under the constraint of controlling the average risk under a pre-specified and clinically meaningful threshold.^56^ Similar to the utility-based approach, the choice of the threshold value is important for making the right decision.

When deriving the optimal ITR, we only use baseline measurements that are routinely collected at the regular donation session. In the future, we may include additional blood-based biomarkers (e.g. those related to iron stores such as ferritin and transferrin) to estimate the optimal donation strategy. Extra costs would be incurred collecting such data, and failure to account for such additional costs can lead to suboptimal decisions from the cost-effectiveness perspective. We will investigate how much additional information on biomarkers adds to the reinforcement of decision-making (compared to the donation strategy estimated solely based on routinely collected data).

Dynamic treatment regimes (DTR) refer to sequential decision rules that adapt over time to the changing status of each subject to maximize the expected long-term target outcomes.^21,57–59^ In the INTERVAL trial, donors were only randomized once at the baseline visit (and then fixed at the initial randomized group), unlike in the sequential multiple assignment randomized trial (SMART) where “treatment assignments” at later decision points are based on the response to the previous randomization and updated individual characteristics.^60^ In addition, we do not have the data on each donor’s Hb levels and iron stores at subsequent donation sessions, even though they may affect the optimal gap time before the next donation. Therefore, based on the data currently available from the INTERVAL trial, we only consider the estimation of single-stage ITRs using baseline measurements in this paper. It would be useful to incorporate dynamic donor stratification and estimate the optimal personalized donation strategy that reflects both heterogeneity across donors and heterogeneity over time within each donor when other data sources become available.

## Supplemental Material

sj-pdf-1-smm-10.1177_0962280220920669 - Supplemental material for Optimal individualized decision rules from a multi-arm trial: A comparison of methods and an application to tailoring inter-donation intervals among blood donors in the UK Supplemental material, sj-pdf-1-smm-10.1177_0962280220920669 for Optimal individualized decision rules from a multi-arm trial: A comparison of methods and an application to tailoring inter-donation intervals among blood donors in the UK by Yuejia Xu, Angela M Wood, Michael J Sweeting, David J Roberts and Brian DM Tom in Statistical Methods in Medical Research
