# Supplementary material for: Optimal individualized decision rules from a multi-arm trial: A comparison of methods and an application to tailoring inter-donation intervals among blood donors in the UK
Source: Stat Methods Med Res. 2020 May 8;29(11):3113–34. doi: 10.1177/0962280220920669 (PMC7682530; doi:10.1177/0962280220920669)
Supplement: sj-pdf-1-smm-10.1177_0962280220920669 - Supplemental material for Optimal individualized decision rules from a multi-arm trial: A comparison of methods and an application to tailoring inter-donation intervals among blood donors in the UK [file sj-pdf-1-smm-10.1177_0962280220920669.pdf]

Statistical Methods in Medical Research
XX(X):1–15
©The Author(s) 2020
Reprints and permission:
sagepub.co.uk/journalsPermissions.nav
DOI: 10.1177/ToBeAssigned
www.sagepub.com/
SAGE

Web-based supplemental materials for “Optimal individualized decision rules from a multi-arm trial: a comparison of methods and an application to tailoring inter-donation intervals among blood donors in the UK” by

Yuejia Xu[1], Angela M. Wood[2,3], Michael J. Sweeting[4,2], David J. Roberts[5,6], and Brian D. M. Tom[1]

Contents

Web Appendix A: quantitative interactions and qualitative interactions 2

Web Appendix B: additional simulation results 3

B.1 Scenarios with correlated covariates . . . 3

B.2 Scenarios with 20 and 50 covariates . . . 4

B.3 Variations of setting 6 (true optimal is the one-size-fits-all rule) . . . 5

Web Appendix C: covariate balance after data cleaning 6

Web Appendix D: additional analysis results based on bootstrap samples 8

Web Appendix E: measure of agreement of estimated ITRs by different methods 10

E.1 The first measure of agreement: percent agreement . . . 10

E.2 The second measure of agreement: pairwise B statistics . . . 11

[1]MRC Biostatistics Unit, University of Cambridge, UK
[2]Cardiovascular Epidemiology Unit, University of Cambridge, UK
[3]NIHR Blood and Transplant Research Unit in Donor Health and Genomics, UK
[4]Department of Health Sciences, University of Leicester, UK
[5]BRC Haematology Theme and Radcliffe Department of Medicine, University of Oxford, UK
[6]National Health Service Blood and Transplant, UK

Corresponding author:
Yuejia Xu, MRC Biostatistics Unit, University of Cambridge, Robinson Way, Cambridge, CB2 0SR, UK
Email: yuejia.xu@mrc-bsu.cam.ac.uk

Web Appendix A: quantitative interactions and qualitative interactions

In Section 4.1. of the main paper, we discuss the distinction between quantitative and qualitative interactions. These two types of interactions are plotted in Supplementary Figure 1.

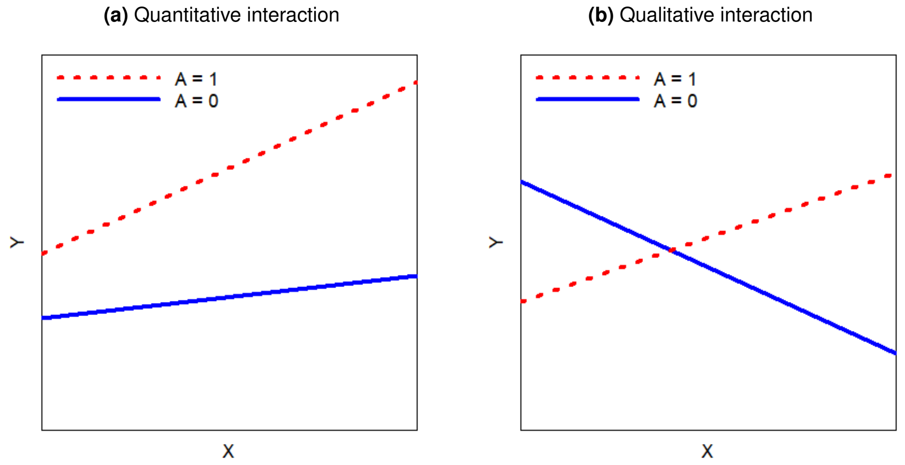

Supplementary Figure 1. Illustration of two types of interactions in the setting with a binary treatment ($K = 2$).

Web Appendix B: additional simulation results

B.1 Scenarios with correlated covariates

We conduct additional simulation studies under scenarios with correlated covariates when $n = 20000$. To demonstrate the idea, we pick settings 1, 2, 3, and 6 from the main paper (settings 1, 2, and 3 correspond to scenarios with tree-type, linear, and nonlinear qualitative treatment-covariate interactions, while setting 6 considers the situation where there is no qualitative interaction and the true optimal treatment is the same for all individuals). Five correlated covariates, $X_1, \ldots, X_5$, are generated. We examine the case with pairwise correlation coefficient being 0.3 and 0.6, respectively. Results are presented in Supplementary Table 1.

Supplementary Table 1. Simulation results under scenarios with correlated covariates based on 100 replicates ($n = 20000$): mean (sd) of misclassification rates and value functions. We examine the case with pairwise correlation between covariates being 0.3 and 0.6. Methods under comparison include the l_1-penalized least squares with hierarchical group LASSO variable selection (l_1-PLS-HGL), l_1-penalized least squares with group LASSO variable selection (l_1-PLS-GL), adaptive contrast weighted learning (ACWL), direct learning (D-learning), and Bayesian additive regression trees (BART). The smallest misclassification rates and the largest value functions for each setting are in bold.

		pairwise correlation = 0.3		pairwise correlation = 0.6	
Setting	Method	Misclassification	Value	Misclassification	Value
1 tree-type qualitative interaction	l_1-PLS-HGL	0.102 (0.009)	1.220 (0.010)	0.074 (0.008)	1.213 (0.012)
	l_1-PLS-GL	0.097 (0.011)	1.223 (0.010)	0.074 (0.008)	1.212 (0.011)
	ACWL	0.080 (0.019)	1.242 (0.010)	0.052 (0.002)	1.243 (0.002)
	D-learning	0.097 (0.012)	1.225 (0.013)	0.075 (0.013)	1.213 (0.017)
	BART	**0.009 (0.004)**	**1.279 (0.003)**	**0.010 (0.005)**	**1.271 (0.004)**
2 linear qualitative interaction	l_1-PLS-HGL	0.020 (0.004)	1.626 (0.003)	0.027 (0.005)	1.534 (0.003)
	l_1-PLS-GL	**0.015 (0.004)**	**1.628 (0.003)**	**0.016 (0.004)**	**1.538 (0.003)**
	ACWL	0.177 (0.017)	1.554 (0.011)	0.170 (0.021)	1.481 (0.009)
	D-learning	0.022 (0.007)	**1.628 (0.003)**	0.023 (0.006)	1.537 (0.004)
	BART	0.063 (0.004)	1.626 (0.007)	0.070 (0.005)	1.532 (0.006)
3 nonlinear qualitative interaction	l_1-PLS-HGL	0.531 (0.016)	1.100 (0.006)	0.445 (0.002)	1.137 (0.003)
	l_1-PLS-GL	0.534 (0.018)	1.097 (0.009)	0.446 (0.005)	1.137 (0.004)
	ACWL	0.529 (0.016)	1.101 (0.006)	0.445 (0.004)	1.133 (0.003)
	D-learning	0.538 (0.024)	1.096 (0.009)	0.453 (0.027)	1.137 (0.006)
	BART	**0.200 (0.045)**	**1.228 (0.012)**	**0.251 (0.030)**	**1.230 (0.010)**
6 tree-type quantitative interaction	l_1-PLS-HGL	**0.000 (0.000)**	**2.114 (0.000)**	**0.000 (0.000)**	**2.114 (0.000)**
	l_1-PLS-GL	**0.000 (0.000)**	**2.114 (0.000)**	**0.000 (0.000)**	**2.114 (0.000)**
	ACWL	**0.000 (0.000)**	**2.114 (0.000)**	**0.000 (0.000)**	**2.114 (0.000)**
	D-learning	**0.000 (0.000)**	**2.114 (0.000)**	**0.000 (0.000)**	**2.114 (0.000)**
	BART	**0.000 (0.000)**	**2.114 (0.000)**	**0.000 (0.000)**	**2.114 (0.000)**

We get similar comparative conclusions across methods as with independent covariates: except for the case of linear decision boundaries (setting 2), BART performs better in all other settings. In addition, when the true optimal is the "one-size-fits-all" rule (setting 6), all methods recover the true optimal ITR with no misclassification.

B.2 Scenarios with 20 and 50 covariates

In this section, we conduct simulation studies under scenarios with more covariates and more prognostic factors than those considered in the main paper. We fix n at 20000. Same as in Section B.1, we pick settings 1, 2, 3 and 6 as representative settings for illustration. In the main paper, we examine the case with $p = 5$ covariates. Here, we run additional simulations with (a) $p = 20$ covariates, among which 10 covariates are involved in the main effect, $m(\mathbf{X}) = 1 + 0.1X_1 - 0.2X_2 - 0.3X_3 + 0.4X_4 - 0.5X_5 + X_6 + X_7 + X_8 - X_9 - X_{10}$, and (b) $p = 50$ covariates, among which 20 covariates are involved in the main effect, $m(\mathbf{X}) = 1 + 0.1X_1 - 0.2X_2 - 0.3X_3 + 0.4X_4 - 0.5X_5 + X_6 + X_7 + X_8 - X_9 - X_{10} + 0.5(X_{11} + X_{12} + X_{13} + X_{14} + X_{15}) - 0.8(X_{16} + X_{17} + X_{18} + X_{19} + X_{20})$. For both (a) and (b), the treatment-covariate interaction effects $\Delta(\mathbf{X}, A)$ remain the same as those in the original settings (described in the main paper Table 1). Results are summarized in Supplementary Table 2:

Supplementary Table 2. Simulation results under scenarios with different numbers of covariates based on 100 replicates ($n = 20000$): mean (sd) of misclassification rates and value functions. We examine the case with $p = 20$ and $p = 50$. Methods under comparison include the l_1-penalized least squares with hierarchical group LASSO variable selection (l_1-PLS-HGL), l_1-penalized least squares with group LASSO variable selection (l_1-PLS-GL), adaptive contrast weighted learning (ACWL), direct learning (D-learning), and Bayesian additive regression trees (BART). The smallest misclassification rates and the largest value functions for each setting are in bold.

		$p = 20$		$p = 50$	
Setting	Method	Misclassification	Value	Misclassification	Value
1 tree-type qualitative interaction	l_1-PLS-HGL	0.108 (0.017)	1.283 (0.015)	0.112 (0.018)	1.267 (0.014)
	l_1-PLS-GL	0.095 (0.014)	1.293 (0.013)	0.101 (0.014)	1.276 (0.013)
	ACWL	0.017 (0.034)	1.348 (0.021)	**0.010 (0.026)**	**1.336 (0.014)**
	D-learning	0.121 (0.019)	1.276 (0.016)	0.134 (0.018)	1.251 (0.017)
	BART	**0.016 (0.007)**	**1.352 (0.005)**	0.030 (0.017)	1.323 (0.011)
2 linear qualitative interaction	l_1-PLS-HGL	**0.015 (0.004)**	1.774 (0.005)	**0.015 (0.003)**	**1.804 (0.005)**
	l_1-PLS-GL	0.020 (0.003)	**1.775 (0.004)**	0.024 (0.004)	1.800 (0.007)
	ACWL	0.175 (0.020)	1.682 (0.022)	0.173 (0.021)	1.712 (0.023)
	D-learning	0.028 (0.007)	1.774 (0.006)	0.036 (0.008)	1.798 (0.007)
	BART	0.066 (0.004)	1.765 (0.009)	0.081 (0.006)	1.777 (0.013)
3 nonlinear qualitative interaction	l_1-PLS-HGL	0.565 (0.006)	1.140 (0.013)	0.567 (0.006)	1.121 (0.020)
	l_1-PLS-GL	0.565 (0.007)	1.136 (0.016)	0.566 (0.005)	1.121 (0.020)
	ACWL	0.559 (0.012)	1.146 (0.011)	0.561 (0.009)	1.120 (0.015)
	D-learning	0.571 (0.009)	1.139 (0.014)	0.574 (0.011)	1.118 (0.020)
	BART	**0.316 (0.053)**	**1.226 (0.017)**	**0.364 (0.027)**	**1.191 (0.022)**
6 tree-type quantitative interaction	l_1-PLS-HGL	**0.000 (0.000)**	**2.151 (0.000)**	**0.000 (0.000)**	**2.123 (0.000)**
	l_1-PLS-GL	**0.000 (0.000)**	**2.151 (0.000)**	**0.000 (0.000)**	**2.123 (0.000)**
	ACWL	**0.000 (0.000)**	**2.151 (0.000)**	**0.000 (0.000)**	**2.123 (0.000)**
	D-learning	**0.000 (0.000)**	**2.151 (0.000)**	**0.000 (0.000)**	**2.123 (0.000)**
	BART	**0.000 (0.000)**	**2.151 (0.000)**	**0.000 (0.000)**	**2.123 (0.000)**

Findings from these moderate-dimensional scenarios are similar to those from low-dimensional scenarios: when decision boundaries are tree-type (setting 1), ACWL and BART perform much better than the other competing methods, while when decision boundaries are linear (setting 2), l_1-PLS-HGL, l_1-PLS-GL and D-learning perform similarly, and significantly better than ACWL as expected. BART outperforms all the other methods under nonlinear decision boundaries (setting 3) due to its flexibility. When the true optimal treatment is the same for everyone (setting 6), all methods perform perfectly with no misclassification. We note that except for BART, all the other methods contain intrinsic variable selection when estimating the optimal ITR (l_1-PLS-HGL, l_1-PLS-GL and D-learning perform LASSO-type variable selection, while ACWL performs variable selection via the node splitting process). However, BART still performs reasonably well for $p = 20$ and $p = 50$ (when $n = 20000$) since we still have $n \gg p$. The performance of BART can be further improved using the variable selection method proposed by Linero[1] for large p at a slightly higher computational cost.

B.3 Variations of setting 6 (true optimal is the one-size-fits-all rule)

In this section, we focus on simulation settings in which the true optimal treatment for everyone is the same (i.e., one-size-fits-all rule) and we examine some variations of setting 6 (of the main paper).

We first consider the case where we increase the noise or reduce the signal in setting 6. We simulate data assuming $Y \sim N(m(\mathbf{X}) + t \times \{I(A=1)\Delta_1(\mathbf{X}) + I(A=2)\Delta_2(\mathbf{X}) + I(A=3)\Delta_3(\mathbf{X})\}, \sigma^2)$. The functional forms of $m(\mathbf{X})$, $\Delta_1(\mathbf{X})$, $\Delta_2(\mathbf{X})$, and $\Delta_3(\mathbf{X})$ are the same as those in setting 6, which implies that treatment 1 is the optimal treatment for everyone. However, in setting 6, we set $t = 0.5$ and $\sigma = 1$, while here we vary values of t and σ with the sample size n fixed at 20000. The results are shown in Supplementary Table 3.

Supplementary Table 3. Simulation results based on 100 replicates ($n = 20000$) under the setting where the optimal ITR is the "one-size-fits-all" rule with different levels of signals and noises: mean (sd) of misclassification rates and value functions. Methods under comparison include the l_1-penalized least squares with hierarchical group LASSO variable selection (l_1-PLS-HGL), l_1-penalized least squares with group LASSO variable selection (l_1-PLS-GL), adaptive contrast weighted learning (ACWL), direct learning (D-learning), and Bayesian additive regression trees (BART). The smallest misclassification rates and the largest value functions for each setting are in bold.

Scenario	Method	Misclassification	Value
$t = 0.5, \sigma = 1$	l_1-PLS-HGL	**0.000 (0.000)**	**2.093 (0.000)**
	l_1-PLS-GL	**0.000 (0.000)**	**2.093 (0.000)**
	ACWL	**0.000 (0.000)**	**2.093 (0.000)**
	D-learning	**0.000 (0.000)**	**2.093 (0.000)**
	BART	**0.000 (0.000)**	**2.093 (0.000)**
$t = 0.5, \sigma = 5$	l_1-PLS-HGL	**0.000 (0.000)**	**1.989 (0.000)**
	l_1-PLS-GL	**0.000 (0.000)**	**1.989 (0.000)**
	ACWL	**0.000 (0.000)**	**1.989 (0.000)**
	D-learning	**0.000 (0.000)**	**1.989 (0.000)**
	BART	0.001 (0.003)	1.988 (0.004)
$t = 0.1, \sigma = 1$	l_1-PLS-HGL	**0.000 (0.000)**	**1.194 (0.000)**
	l_1-PLS-GL	**0.000 (0.000)**	**1.194 (0.000)**
	ACWL	**0.000 (0.000)**	**1.194 (0.000)**
	D-learning	**0.000 (0.000)**	**1.194 (0.000)**
	BART	0.001 (0.003)	**1.194 (0.001)**

We observe that when the noise level increases to $\sigma = 5$, all methods still perform perfectly in the setting where the "one-size-fits-all" rule is the optimal treatment regime. When we fix σ and reduce the value of t from 0.5 to 0.1, the perfect classification results remain. These sensitivity analysis results are not surprising since the sample size we consider is sufficiently large.

In setting 6, the quantitative interactions are tree-type. We also consider other types of quantitative interactions, such as linear additive and nonlinear (Supplementary Table 4).

According to Supplementary Table 4, when the true optimal treatment is the same for all subjects ("trivial" decision rule that assigns all to the marginally best treatment), all methods perform perfectly with no misclassification, regardless of whether the type of underlying quantitative interactions are linear, nonlinear, or tree-type.

Supplementary Table 4. Simulation results based on 100 replicates ($n = 20000$) under the setting where the optimal ITR is the "one-size-fits-all" rule with different types of quantitative interactions: mean (sd) of misclassification rates and value functions. Methods under comparison include the l_1-penalized least squares with hierarchical group LASSO variable selection (l_1-PLS-HGL), l_1-penalized least squares with group LASSO variable selection (l_1-PLS-GL), adaptive contrast weighted learning (ACWL), direct learning (D-learning), and Bayesian additive regression trees (BART). The smallest misclassification rates and the largest value functions for each setting are in bold.

Forms of quantitative interactions	Method	Misclassification	Value
tree	l_1-PLS-HGL	**0.000 (0.000)**	**2.093 (0.000)**
$\Delta_1(\mathbf{X}) = I(X_1 > 0.5) + 2$	l_1-PLS-GL	**0.000 (0.000)**	**2.093 (0.000)**
$\Delta_2(\mathbf{X}) = 2 \times I(X_2 \geq 0.5)I(X_3 < 0.25) - 3$	ACWL	**0.000 (0.000)**	**2.093 (0.000)**
	D-learning	**0.000 (0.000)**	**2.093 (0.000)**
$\Delta_3(\mathbf{X}) = 0$	BART	**0.000 (0.000)**	**2.093 (0.000)**
linear	l_1-PLS-HGL	**0.000 (0.000)**	**3.975 (0.000)**
$\Delta_1(\mathbf{X}) = 3X_1 - 2X_2 + 6$	l_1-PLS-GL	**0.000 (0.000)**	**3.975 (0.000)**
$\Delta_2(\mathbf{X}) = 5X_3 - X_4 + X_5 - 8$	ACWL	**0.000 (0.000)**	**3.975 (0.000)**
	D-learning	**0.000 (0.000)**	**3.975 (0.000)**
$\Delta_3(\mathbf{X}) = 0$	BART	**0.000 (0.000)**	**3.975 (0.000)**
nonlinear	l_1-PLS-HGL	**0.000 (0.000)**	**2.386 (0.000)**
$\Delta_1(\mathbf{X}) = 3X_1^2 - \exp(X_2) + 3$	l_1-PLS-GL	**0.000 (0.000)**	**2.386 (0.000)**
$\Delta_2(\mathbf{X}) = X_3^3 - 2$	ACWL	**0.000 (0.000)**	**2.386 (0.000)**
	D-learning	**0.000 (0.000)**	**2.386 (0.000)**
$\Delta_3(\mathbf{X}) = 0$	BART	**0.000 (0.000)**	**2.386 (0.000)**

Web Appendix C: covariate balance after data cleaning

In this section, we examine the covariate balance after data cleaning, and we present donors' baseline characteristics by randomized groups (inter-donation intervals) after data cleaning in Supplementary Table 5 for continuous covariates and Supplementary Table 6 for categorical covariates, respectively. Based on these results, we conclude that the data cleaning process does not distort the balance of baseline covariates across randomized groups.

Supplementary Table 5. Mean (sd) of continuous baseline characteristics by randomized groups after data cleaning (rounded to 3 significant digits).

Baseline covariates	8-week	10-week	12-week
Age (years)	45.7 (14.0)	45.7 (14.2)	45.8 (14.0)
Body mass index (kg/m^2)	26.8 (6.74)	26.7 (5.42)	26.7 (5.65)
SF-36v2 physical component score	56.8 (4.57)	56.9 (4.49)	56.8 (4.49)
SF-36v2 mental component score	54.7 (5.90)	54.6 (6.19)	54.6 (5.99)
Blood donations in the 2 years before trial enrollment	3.64 (1.85)	3.66 (1.84)	3.65 (1.83)
Haemoglobin level (g/dL)	15.0 (1.01)	15.0 (0.992)	15.0 (0.987)
White blood cell count (10^9/L)	6.15 (1.50)	6.15 (1.52)	6.15 (1.51)
Red blood cell count (10^{12}/L)	5.05 (0.383)	5.04 (0.381)	5.04 (0.381)
Mean corpuscular haemoglobin (pg)	29.7 (1.72)	29.7 (1.67)	29.7 (1.66)
Mean corpuscular volume (fL)	92.2 (4.64)	92.3 (4.64)	92.3 (4.67)
Platelet count (10^9/L)	229 (49.7)	229 (50.2)	228 (50.7)

Supplementary Table 6. Number of participants (percentages) of categorical baseline characteristics by randomized groups after data cleaning.

Baseline covariates	8-week	10-week	12-week
Ethnicity			
White	5790 (83.8%)	5751 (83.8%)	5675 (83.4%)
Black	48 (0.7%)	50 (0.7%)	59 (0.9%)
Asian	161 (2.3%)	163 (2.4%)	180 (2.6%)
Mixed	84 (1.2%)	64 (0.9%)	71 (1.0%)
Other	32 (0.5%)	16 (0.2%)	27 (0.4%)
Unknown	796 (11.5%)	815 (11.9%)	792 (11.6%)
Blood group			
A-	518 (7.5%)	523 (7.6%)	544 (8.0%)
A+	2137 (30.9%)	2112 (30.8%)	2122 (31.2%)
AB-	46 (0.7%)	59 (0.9%)	48 (0.7%)
AB+	208 (3.0%)	171 (2.5%)	187 (2.7%)
B-	130 (1.9%)	128 (1.9%)	166 (2.4%)
B+	597 (8.6%)	590 (8.6%)	566 (8.3%)
O-	789 (11.4%)	734 (10.7%)	729 (10.7%)
O+	2486 (36.0%)	2542 (37.1%)	2442 (35.9%)
Iron prescription			
Yes	8 (0.1%)	13 (0.2%)	26 (0.4%)
No	6833 (98.9%)	6767 (98.7%)	6692 (98.4%)
Unknown	70 (1.0%)	79 (1.2%)	86 (1.3%)
Smoke ever			
Yes	2867 (41.5%)	2824 (41.2%)	2870 (42.2%)
No	3989 (57.7%)	3980 (58.0%)	3869 (56.9%)
Unknown	55 (0.8%)	55 (0.8%)	65 (1.0%)
Smoke currently			
Yes	483 (2.3%)	566 (2.8%)	520 (2.5%)
No	2368 (11.5%)	2239 (10.9%)	2319 (11.3%)
Unknown	4060 (19.7%)	4054 (19.7%)	3965 (19.3%)
Alcohol ever			
Yes	6716 (97.2%)	6676 (97.3%)	6608 (97.1%)
No	176 (2.5%)	167 (2.4%)	161 (2.4%)
Unknown	19 (0.3%)	16 (0.2%)	35 (0.5%)
Alcohol currently			
Yes	6060 (29.5%)	6026 (29.3%)	5979 (29.1%)
No	421 (2.0%)	421 (2.0%)	380 (1.8%)
Unknown	430 (2.1%)	412 (2.0%)	445 (2.2%)
New or returning donor status			
New	509 (7.4%)	493 (7.2%)	484 (7.1%)
Returning	6402 (92.6%)	6366 (92.8%)	6320 (92.9%)

Web Appendix D: additional analysis results based on bootstrap samples

In Tables 2-4 of the main paper, we present means and standard deviations of donor assignment proportions and empirical ITR effects across 100 repetitions of 5-fold cross-validation. The standard deviation estimates based on cross-validation reflect the repeatability of a method, i.e., how much variation would we expect in the answers obtained if we employ the method with cross-validation to the same dataset multiple times. In this section, we present additional analysis results based on 500 bootstrap samples (stratified by randomized groups) from male donors in the INTERVAL trial (Supplementary Tables 7-10). Different from the cross-validation approach, the bootstrap approach attempts to capture the confidence we have in the size of the effects estimated by different methods, i.e., how much variation would we expect in the effect estimates if we were to repeat the study multiple times in the same population and calculate an effect estimate using the same method each time.

Supplementary Table 7. Applications of the l_1-penalized least squares with hierarchical group LASSO variable selection (l_1-PLS-HGL), l_1-penalized least squares with group LASSO variable selection (l_1-PLS-GL), adaptive contrast weighted learning (ACWL), and direct learning (D-learning) to data from male donors in the INTERVAL trial. Sample estimates based on the INTERVAL data and standard deviation estimates based on 500 bootstrap samples (in parenthesis) of assignment proportions in % and empirical ITR effects on donation and deferral outcomes are reported. ITR effects measure the difference in the average outcome between donors whose assigned inter-donation intervals in the trial are optimal (with respect to the method used to estimate the ITR) and those whose assigned inter-donation intervals are non-optimal. A larger ITR effect on donation and a smaller ITR effect on deferral are more desirable. The first four and last four rows correspond to the target being maximizing total units of blood collected by the blood service, and minimizing the rate of low Hb deferrals, respectively.

		Assignment Percentages			ITR Effects	
Target Outcome	Method	12 weeks	10 weeks	8 weeks	Donation	Deferral
Donation	l_1-PLS-HGL	0.1 (0.2)	0.2 (0.7)	99.7 (0.7)	1.313 (0.047)	0.026 (0.002)
	l_1-PLS-GL	0.1 (0.2)	1.9 (1.1)	98.1 (1.2)	1.326 (0.048)	0.025 (0.002)
	ACWL	0.0 (0.0)	0.0 (0.4)	100.0 (0.4)	1.315 (0.048)	0.027 (0.002)
	D-learning	0.5 (1.1)	0.7 (2.9)	98.8 (3.1)	1.310 (0.063)	0.026 (0.002)
Deferral	l_1-PLS-HGL	93.1 (2.6)	6.8 (2.6)	0.0 (0.2)	-1.191 (0.050)	-0.024 (0.002)
	l_1-PLS-GL	100.0 (6.1)	0.0 (5.7)	0.0 (0.5)	-1.248 (0.083)	-0.025 (0.002)
	ACWL	100.0 (3.1)	0.0 (3.1)	0.0 (0.2)	-1.248 (0.053)	-0.025 (0.002)
	D-learning	94.9 (4.0)	4.9 (4.1)	0.2 (0.8)	-1.197 (0.067)	-0.024 (0.002)

Supplementary Table 8. Sample estimates based on the INTERVAL data and standard deviation estimates based on 500 bootstrap samples (in parenthesis) of empirical ITR effects of three non-personalized rules on donation and deferral outcomes. ITR effects measure the difference in the average outcome between donors whose assigned inter-donation intervals in the trial are the same as the one specified in the non-personalized rule and those whose assigned inter-donation intervals are different from that specified in the non-personalized rule. A larger ITR effect on donation and a smaller ITR effect on deferral are more desirable.

	ITR Effects	
Non-personalized Rule	Donation	Deferral
Recommend all male donors to donate every 12 weeks	-1.248 (0.040)	-0.025 (0.002)
Recommend all male donors to donate every 10 weeks	-0.077 (0.042)	-0.002 (0.002)
Recommend all male donors to donate every 8 weeks	1.315 (0.048)	0.027 (0.002)

Supplementary Table 7 presents the results for analyzing two outcomes separately. Supplementary Table 8 shows empirical ITR effects of three non-personalized rules on both the donation and the deferral outcome.

Supplementary Table 9. Applications of the l_1-penalized least squares with hierarchical group LASSO variable selection (l_1-PLS-HGL), l_1-penalized least squares with group LASSO variable selection (l_1-PLS-GL), adaptive contrast weighted learning (ACWL), and direct learning (D-learning) to data from male donors in the INTERVAL trial assuming the target is to maximize the utility. The trade-off parameter b in the utility function varies from 1 to 5 at an increment of 1. Sample estimates based on the INTERVAL data and standard deviation estimates based on 500 bootstrap samples (in parenthesis) of assignment proportions in % and empirical ITR effects on donation, deferral, and utility are reported. ITR effects measure the difference in the average outcome between donors whose assigned inter-donation intervals in the trial are optimal (with respect to the method used to estimate the ITR) and those whose assigned inter-donation intervals are non-optimal. A larger ITR effect on donation/utility and a smaller ITR effect on deferral are more desirable.

		Assignment Percentages			ITR Effects		
Trade-off Parameter	Method	12 weeks	10 weeks	8 weeks	Donation	Deferral	Utility
$b = 1$	l_1-PLS-HGL	0.9 (0.6)	1.0 (1.7)	98.1 (1.7)	1.331 (0.050)	0.022 (0.002)	1.089 (0.050)
	l_1-PLS-GL	0.5 (0.4)	3.8 (1.9)	95.7 (2.0)	1.320 (0.050)	0.023 (0.002)	1.079 (0.049)
	ACWL	0.0 (0.1)	0.0 (1.9)	100.0 (1.9)	1.315 (0.048)	0.027 (0.002)	1.055 (0.050)
	D-learning	0.7 (1.5)	2.6 (4.2)	96.7 (4.5)	1.323 (0.076)	0.024 (0.003)	1.079 (0.065)
$b = 2$	l_1-PLS-HGL	3.9 (1.3)	3.8 (3.3)	92.2 (3.1)	1.277 (0.061)	0.019 (0.002)	0.869 (0.055)
	l_1-PLS-GL	0.0 (1.0)	0.8 (3.6)	99.2 (3.8)	1.320 (0.061)	0.026 (0.003)	0.806 (0.055)
	ACWL	3.7 (2.9)	0.0 (5.9)	96.3 (5.0)	1.299 (0.061)	0.022 (0.003)	0.838 (0.054)
	D-learning	0.6 (2.3)	6.8 (5.6)	92.6 (5.9)	1.304 (0.092)	0.022 (0.003)	0.863 (0.064)
$b = 3$	l_1-PLS-HGL	9.8 (2.6)	11.4 (5.3)	78.9 (4.6)	1.122 (0.079)	0.009 (0.003)	0.767 (0.061)
	l_1-PLS-GL	6.6 (2.3)	19.0 (5.7)	74.4 (6.6)	1.104 (0.087)	0.011 (0.004)	0.744 (0.072)
	ACWL	14.8 (4.2)	12.4 (10.6)	72.9 (9.5)	1.009 (0.119)	0.007 (0.004)	0.731 (0.066)
	D-learning	6.2 (3.4)	13.7 (5.9)	80.1 (5.7)	1.087 (0.096)	0.014 (0.004)	0.659 (0.069)
$b = 4$	l_1-PLS-HGL	18.1 (3.7)	22.2 (6.7)	59.7 (5.2)	0.816 (0.092)	-0.002 (0.003)	0.765 (0.067)
	l_1-PLS-GL	14.1 (4.1)	30.7 (6.6)	55.2 (6.7)	0.810 (0.106)	-0.001 (0.004)	0.756 (0.101)
	ACWL	15.7 (7.5)	9.5 (14.1)	74.9 (12.0)	0.931 (0.168)	0.007 (0.006)	0.560 (0.085)
	D-learning	15.7 (4.4)	27.1 (5.9)	57.1 (5.2)	0.740 (0.100)	0.000 (0.004)	0.667 (0.075)
$b = 5$	l_1-PLS-HGL	27.2 (4.5)	32.3 (7.2)	40.5 (5.2)	0.453 (0.098)	-0.009 (0.003)	0.751 (0.073)
	l_1-PLS-GL	23.5 (5.8)	39.3 (8.8)	37.2 (5.6)	0.486 (0.104)	-0.010 (0.004)	0.810 (0.121)
	ACWL	30.3 (8.0)	16.2 (15.1)	53.5 (12.2)	0.522 (0.182)	-0.004 (0.005)	0.579 (0.097)
	D-learning	19.3 (4.7)	36.8 (6.3)	44.0 (5.0)	0.587 (0.102)	-0.006 (0.003)	0.766 (0.078)

Supplementary Table 9 summarizes allocation proportions and ITR effects when the aim is to maximize the utility score with different values of the trade-off parameter b, and Supplementary Table 10 presents the ITR effects of three non-personalized rules on the utility score when the trade-off parameter b varies from 1 to 5 at an increment of 1. All point estimates presented in Supplementary Tables 7-10 are corresponding sample statistics computed from the original INTERVAL data, and standard deviation estimates (in parenthesis) are calculated based on 500 bootstrap samples.[2]

Results on assignment percentages and ITR effects indicate that different methods perform similarly. Not surprisingly, bootstrap-based standard deviation estimates that reflect the uncertainty of the observed dataset are much larger than cross-validation-based standard deviation estimates reported in the main paper.

Supplementary Table 10. Sample estimates based on the INTERVAL data and standard deviation estimates based on 500 bootstrap samples (in parenthesis) of ITR effects of three non-personalized rules on the utility outcome. The trade-off parameter b in the utility function varies from 1 to 5 at an increment of 1. ITR effects measure the difference in the average outcome between donors whose assigned inter-donation intervals in the trial are the same as the one specified in the non-personalized rule and those whose assigned inter-donation intervals are different from that specified in the non-personalized rule. A larger ITR effect on utility is more desirable.

	ITR Effects on Utility				
Non-personalized Rule	$b=1$	$b=2$	$b=3$	$b=4$	$b=5$
Recommend all male donors to donate every 12 weeks	-1.038 (0.042)	-0.828 (0.045)	-0.618 (0.049)	-0.408 (0.055)	-0.199 (0.060)
Recommend all male donors to donate every 10 weeks	-0.025 (0.044)	0.027 (0.048)	0.079 (0.053)	0.131 (0.058)	0.183 (0.065)
Recommend all male donors to donate every 8 weeks	1.055 (0.050)	0.795 (0.054)	0.535 (0.059)	0.275 (0.066)	0.015 (0.074)

Web Appendix E: measure of agreement of estimated ITRs by different methods

Results presented in Tables 2 and 3 in the main paper indicate that donor assignment proportions and empirical ITR effects seem to be very similar across different methods for a given target outcome. In this section, we examine the degree of agreement between different methods in terms of the estimated optimal inter-donation interval for each male donor in the INTERVAL trial. We present two types of agreement measures.

E.1 The first measure of agreement: percent agreement

The first one is a simple "agreement proportion" measure that assesses the amount of "overlap" of the optimal decisions made according to each method (l_1-PLS-HGL, l_1-PLS-GL, ACWL, D-learning, and BART). We calculate the "observed proportion of agreement" (percentage of male donors who receive the same recommendation on his optimal inter-donation interval by all five methods):

$$\frac{\sum_{i=1}^{N} I\Big[\max\big\{\sum_{j=1}^{5} I(\widehat{\mathcal{D}_j^*}(\mathbf{X}_i)=8\text{ weeks}),\sum_{j=1}^{5} I(\widehat{\mathcal{D}_j^*}(\mathbf{X}_i)=10\text{ weeks}),\sum_{j=1}^{5} I(\widehat{\mathcal{D}_j^*}(\mathbf{X}_i)=12\text{ weeks})\big\}=5\Big]}{N},$$

where max(.) is the maximum function, $I(.)$ is the indicator function, i is the donor index, $i=1,\dots,N$, j is the method index, $j=1,\dots,5$, and $\widehat{\mathcal{D}_j^*}(\mathbf{X}_i)$ denotes the optimal inter-donation interval estimated by method j for the ith donor.

In the case where recommendations are not consistent across all five methods, but three or four out of five methods "agree", the majority voting rule can be used to determine the optimal individualized inter-donation interval. Therefore, we also calculate the percentage of male donors of whom at least four methods "agree" on his optimal inter-donation interval:

$$\frac{\sum_{i=1}^{N} I\Big[\max\big\{\sum_{j=1}^{5} I(\widehat{\mathcal{D}_j^*}(\mathbf{X}_i)=8\text{ weeks}),\sum_{j=1}^{5} I(\widehat{\mathcal{D}_j^*}(\mathbf{X}_i)=10\text{ weeks}),\sum_{j=1}^{5} I(\widehat{\mathcal{D}_j^*}(\mathbf{X}_i)=12\text{ weeks})\big\}\geq 4\Big]}{N},$$

and the percentage of male donors of whom at least three methods "agree" on his optimal inter-donation interval:

$$\frac{\sum_{i=1}^{N} I\Big[\max\big\{\sum_{j=1}^{5} I(\widehat{\mathcal{D}_j^*}(\mathbf{X}_i)=8\text{ weeks}),\sum_{j=1}^{5} I(\widehat{\mathcal{D}_j^*}(\mathbf{X}_i)=10\text{ weeks}),\sum_{j=1}^{5} I(\widehat{\mathcal{D}_j^*}(\mathbf{X}_i)=12\text{ weeks})\big\}\geq 3\Big]}{N}.$$

Results are presented in Supplementary Table 11.

We observe that when the target outcome is donation, deferral, or utility with $b=1$ or 2, the levels of agreement between five methods are high ($>80\%$). As the trade-off parameter b in the utility function

Supplementary Table 11. Percentages of male donors who receive the same recommendation on the optimal inter-donation interval by all five methods, by at least four methods, or by at least three methods when the target outcome is donation, deferral, and utility, respectively. Methods to estimate the optimal individualized inter-donation interval include the l_1-penalized least squares with hierarchical group LASSO variable selection, l_1-penalized least squares with group LASSO variable selection, adaptive contrast weighted learning, direct learning, and Bayesian additive regression trees.

Agreement Type	Donation	Deferral	Utility				
			$b=1$	$b=2$	$b=3$	$b=4$	$b=5$
All Five Methods Agree	97.5	86.9	92.2	82.6	59.0	45.2	33.5
At Least Four Methods Agree	99.1	94.4	96.9	92.5	79.0	71.2	64.3
At Least Three Methods Agree	100.0	100.0	99.5	97.8	96.3	95.5	94.4

increases, the levels of agreement decrease. For the most stringent agreement criterion (regarded as "agree" only if all five methods lead to the same recommendation), the percentage of donors getting the same recommendation from all five methods is only 33.5% for $b = 5$. On the other hand, according to the least stringent criterion of agreement (regarded as "agree" if at least three methods agree), we can always decide the optimal inter-donation interval for at least 90% of donors using the majority voting rule regardless of the target outcome.

E.2 The second measure of agreement: pairwise B statistics

One limitation of the "percent agreement" measure is that this type of statistics does not account and correct for the "agreement by chance". Chance-corrected measures have been proposed for assessing inter-rater agreement, for example, the Cohen's kappa for two raters or Fleiss' kappa for more than two raters.[3,4] However, we note that kappa is not particularly suitable in the current setting because the performance of kappa depends on marginal distributions, while in our case, marginal distributions are highly symmetrically-imbalanced, especially when the target outcome is donation, deferral, or utility with small values for b. This phenomenon has been well-documented in the literature and is commonly referred to as the "high agreement but low kappa paradox".[5–7] Some improved measures that still account for chance agreement but address the kappa paradox have been proposed. For example, the Bangdiwala's B statistics proposed by Bangdiwala[8] has been shown to be robust to different marginal distributions.[7,9] However, B statistics has not been extended to handle the case with more than two raters. We report the pairwise B statistics as the second type of agreement measure and use the guidelines suggested by Munoz and Bangdiwala[10] for the interpretation of B statistics (Supplementary Table 12).

Supplementary Figure 2 presents the pairwise B statistics among 5 methods when the donation and deferral outcomes are analyzed separately, and Supplementary Figure 3 shows the corresponding results when the utility score is the target outcome. Different colors indicate the extent of agreement according to pairwise B statistics with yellow or light yellow representing the situations where the agreement is "almost perfect" or "perfect". We observe that all pairwise B statistics suggest at least "almost perfect" agreements when the target outcome is donation, deferral, or utility with $b = 1$ or 2. As b gets larger, the degree of pairwise agreements decreases. For $b = 3$ or 4, pairwise agreements never go below "substantial", whereas when the target outcome is the utility score with $b = 5$, several pairwise B statistics indicate only "moderate" agreement. In general, pairwise B statistics between l_1-PLS-HGL and the other methods seem to be slightly higher than the rest of

Supplementary Table 12. Guidelines for the interpretation of values of B statistics.[10]

Range of B statistics	Agreement Level
[0,0.09)	Poor agreement
[0.09,0.25)	Fair agreement
[0.25,0.49)	Moderate agreement
[0.49,0.81)	Substantial agreement
[0.81,1.00)	Almost perfect agreement
1.00	Perfect agreement

the pairwise B statistics. Even though l_1-PLS-HGL and l_1-PLS-GL are built under the same framework and the only difference between these two methods is the variable selection approach used when constructing the outcome model, the levels of agreement between these two methods do not seem to be consistently high. In cases where estimated optimal ITRs are far from the "one-size-fits-all" regime (e.g. when the target outcome is the utility score with $b = 3$, 4, or 5), pairwise agreements between BART and l_1-PLS-HGL are higher than those between BART and ACWL. This may be due to the fact that both l_1-PLS-HGL and BART use a two-stage procedure to estimate the optimal ITR, where in the first stage, the outcome model is constructed, and in the second stage, the optimal ITR is derived as the one that optimizes the expected outcome. On the other hand, even though both ACWL and BART are tree-based methods, BART uses "additive regression trees" to model the dependency structure between the response and covariates, whereas ACWL uses "classification trees" to solve the weighted classification problem and estimate the decision rule. We also observe that in general, the pairwise B statistics between BART and other methods are lower than the rest of the pairwise B statistics, especially when the aim is to maximize the utility score with $b = 3$, 4, or 5.

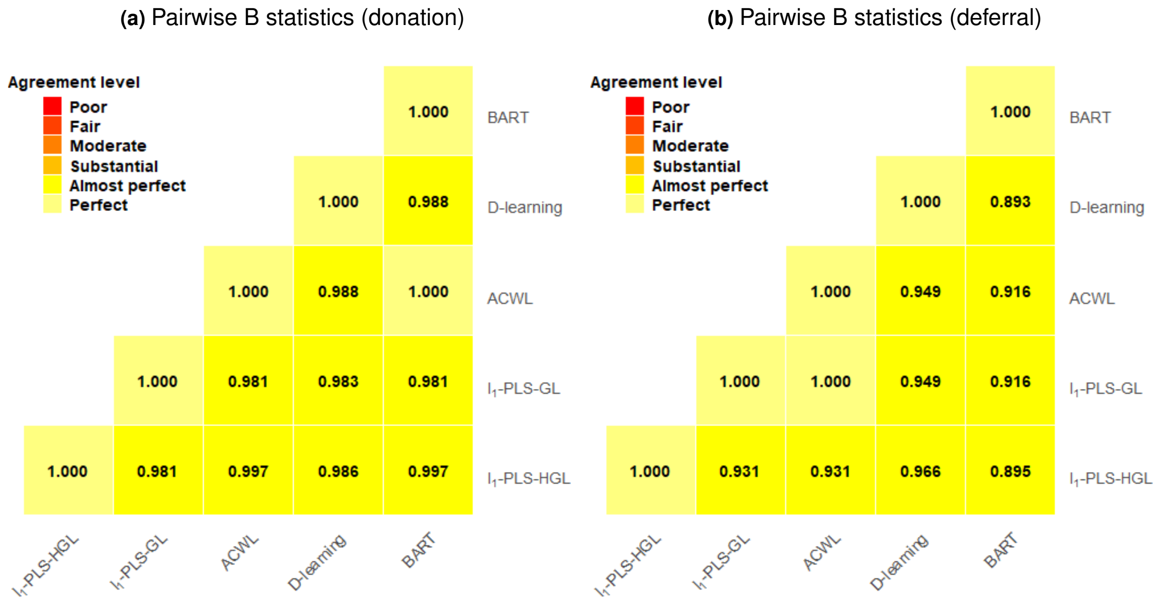

Supplementary Figure 2. Pairwise B statistics that measure the agreement of the optimal individualized donation strategies estimated using five different methods: l_1-penalized least squares with hierarchical group LASSO variable selection (l_1-PLS-HGL), l_1-penalized least squares with group LASSO variable selection (l_1-PLS-GL), adaptive contrast weighted learning (ACWL), direct learning (D-learning), and Bayesian additive regression trees (BART). Decision rules are obtained with the aim of (a) maximizing the units of blood collected by the blood service, and (b) minimizing the low Hb deferral rates using the data from male donors in the INTERVAL trial. Different colors indicate different levels of agreement.

(a) Pairwise B statistics (utility with $b = 1$)

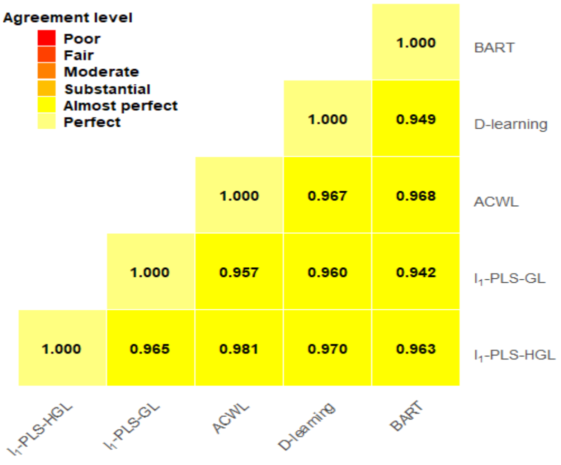

(b) Pairwise B statistics (utility with $b = 2$)

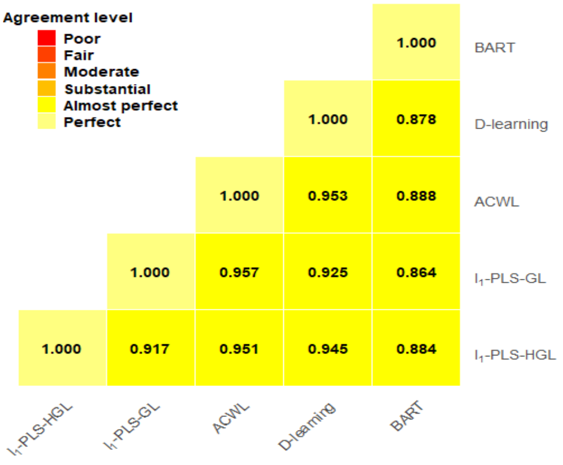

(c) Pairwise B statistics (utility with $b = 3$)

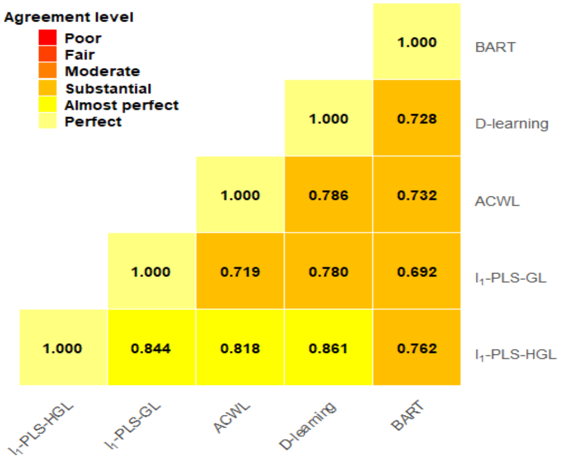

(d) Pairwise B statistics (utility with $b = 4$)

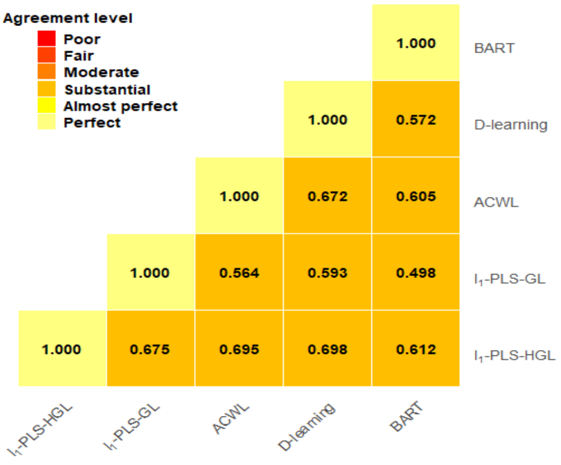

(e) Pairwise B statistics (utility with $b = 5$)

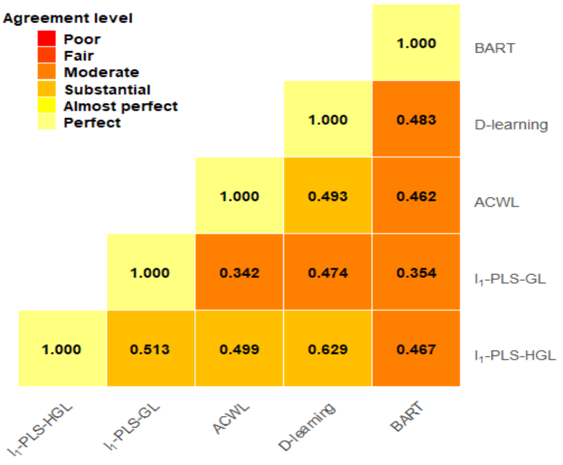

Supplementary Figure 3. Pairwise B statistics that measure the agreement of the optimal individualized donation strategies estimated using five different methods: l_1-penalized least squares with hierarchical group LASSO variable selection (l_1-PLS-HGL), l_1-penalized least squares with group LASSO variable selection (l_1-PLS-GL), adaptive contrast weighted learning (ACWL), direct learning (D-learning), and Bayesian additive regression trees (BART). Decision rules are obtained with the aim of maximizing the utility score using the data from male donors in the INTERVAL trial. The trade-off parameter b in the utility function varies from 1 to 5 at an increment of 1. Different colors indicate different levels of agreement.

References

1. Linero AR. Bayesian regression trees for high-dimensional prediction and variable selection. *J Am Stat Assoc* 2018; 113(522): 626–636.
2. Efron B and Tibshirani RJ. *An Introduction to the Bootstrap*. Number 57 in Monographs on Statistics and Applied Probability, Boca Raton, Florida, USA: Chapman & Hall/CRC, 1993.
3. Cohen J. A coefficient of agreement for nominal scales. *Educ Psychol Meas* 1960; 20(1): 37–46.
4. Fleiss J. Measuring nominal scale agreement among many raters. *Psychol Bull* 1971; 76(5): 378–382.
5. Feinstein AR and Cicchetti DV. High agreement but low kappa: I. the problems of two paradoxes. *J Clin Epidemiol* 1990; 43(6): 543 – 549.
6. Gwet KL. Computing inter-rater reliability and its variance in the presence of high agreement. *Brit J Math Stat Psychol* 2008; 61(1): 29–48.
7. Shankar V and Bangdiwala SI. Observer agreement paradoxes in 2x2 tables: comparison of agreement measures. *BMC Med Res Methodol* 2014; 14(1): Article 100.
8. Bangdiwala SI. A graphical test for observer agreement. In *Proceedings of the 45th International Statistical Institute Meeting*. Amsterdam, 1985. pp. 307–308.
9. Shankar V and Bangdiwala SI. Behavior of agreement measures in the presence of zero cells and biased marginal distributions. *J Appl Stat* 2008; 35(4): 445–464.
10. Munoz SR and Bangdiwala SI. Interpretation of kappa and B statistics measures of agreement. *J Appl Stat* 1997; 24(1): 105–112.
